# The extracellular matrix in inflammation and cancer

**DOI:** 10.1186/s43556-026-00415-6

**Published:** 2026-03-06

**Authors:** Wanting Zhang, Yuhang Xiang, Chen Lu, Fei Wang, He Ren, Hao Wu, Meisi Yan

**Affiliations:** 1 Department of Pathology, School of Basic Medical Sciences, Harbin Medical University, Harbin, Heilongjiang Province 150081 China; 2 Department of Oncological Surgery, Harbin Medical University Cancer Hospital, Harbin, Heilongjiang Province China; 3 Heilongjiang Clinical Research Center for Breast Cancer, Harbin Medical University Cancer Hospital, Harbin, China; 4 Genomics Research Center, State Key Laboratory of Biomedicine-Pharmaceutics of China, College of Pharmacy, Harbin Medical University, Harbin, China

**Keywords:** Extracellular matrix, Immune response, Extracellular matrix remodeling, Inflammation, Therapeutic strategies

## Abstract

The extracellular matrix (ECM) forms a dynamic structure around cells, providing environmental cues, mechanical support, and tissue protection. It is composed of fibrous proteins, glycosaminoglycans (GAGs), proteoglycans, and glycoproteins. The molecular, physical, and mechanical properties of the ECM regulate the motility, survival, and function of immune cells. In most cancers, inflammatory cytokines and proteases—particularly matrix metalloproteinases(MMPs)—released within the immune-infiltrated inflammatory microenvironment can remodel the ECM. Cytokines such as tumor necrosis factor-α (TNF), interleukin, and transforming growth factor-beta (TGF-β) modulate the expression of various ECM molecules and promote host cell differentiation, thereby shaping a stroma conducive to tumor survival and metastasis. When ECM components become dysregulated, they act as ligands interacting with immune cell receptors, suppressing the function of specific immune cell subsets in the tumor microenvironment (TME), and activating downstream intracellular signaling pathways that are exploited by cancer cells to facilitate progression. This review systematically outlines key ECM constituents, molecular mediators of ECM remodeling, and their role in regulating immune cell behavior, including T cell exhaustion and macrophage polarization. It also elucidates the direct interactions between ECM and immune cells within inflammatory settings. Furthermore, we explore therapeutic strategies targeting ECM-mediated immunosuppression in solid tumors. This study highlights promising approaches to enhance the efficacy of cancer immunotherapy.

## Introduction

Cancer progression, persistent inflammation in the surrounding tissues, and ECM remodeling are three highly interconnected processes. Indeed, the multifaceted inflammatory signaling network generated by cancer cells and innate immune cells within the TME induces changes in the surrounding stroma, which in turn disrupts ECM homeostasis. This process fosters a "cancerized" microenvironment that supports tumor growth and metastasis [1]. In recent years, with in-depth research on solid tumors, the TME has garnered increasing attention owing to its critical role in tumor progression, local drug resistance, immune suppression, and targeted therapy [2–5]. The TME is composed of diverse cellular and non-cellular components that collectively drive tumor growth, invasion, metastasis, and treatment response [6]. Cellular components include cancer-associated fibroblasts (CAFs), endothelial cells (ECs), epithelial cells, and immune cells, whereas the non-cellular compartment consists of the ECM [7].

The ECM is a dynamic three-dimensional macromolecular network that provides structural support to tissues and cells, playing essential structural and functional roles in both tissue remodeling and the regulation of cellular processes [8]. From a molecular composition perspective, the ECM primarily comprises structural proteins, such as collagen, elastin, and fibronectin (FN), as well as non-structural molecules, such as proteoglycans and hyaluronan (HA) [9]. ECM remodeling is accompanied by dysregulated immune activation and poses a significant physical barrier to effective immune modulation by impeding the entry of immune cells and therapeutics into the tissues of interest [10]. Increased ECM stiffness within the TME is largely driven by ECM remodeling. ECM stiffness is an important biomechanical property that reflects the resistance of the matrix to deformation under mechanical stress. It significantly influences immune cell activation, polarization, migration, infiltration, cytotoxicity, and antigen presentation, thereby profoundly affecting the efficacy of tumor immunotherapy. A key aspect of the TME is intercellular crosstalk and cell-ECM communication, interactions that promote immune evasion and further contribute to therapy resistance [11]. By acting as ligands for cell surface receptors, ECM components continuously interact with resident cells such as fibroblasts and immune cells and transmit signals that regulate adhesion, migration, proliferation, apoptosis, and survival. This process is highly complex and requires precise control to maintain tissue homeostasis [12].

In this review, we aimed to outline the role of the ECM in tumor progression, including the impact of inflammation-associated secretory factors on alterations in the composition of the ECM. We also discuss the contributions of molecular mediators and immune cells to ECM remodeling. A remodeled ECM not only creates a physical barrier that impedes immune cell infiltration and drug delivery but also engages in molecular crosstalk with immune cells through receptors such as integrins, discoid domain receptors (DDR), leukocyte-associated Ig-like receptors (LAIR-1), and cluster of differentiation 44 (CD44). Although the role of the ECM in shaping the immunosuppressive niche is increasingly being recognized, a comprehensive understanding of how specific ECM components and biomechanical properties coordinately regulate immune cell functions across different cancer types remains limited. Moreover, although ECM-targeting therapeutic strategies have shown promise in preclinical models, their clinical translation is hindered by challenges such as tumor heterogeneity, off-target effects, and the complexity of ECM-immune interactions. This review seeks to systematically integrate the current knowledge on ECM structure, remodeling mechanisms, and immune regulation to identify novel therapeutic avenues for overcoming ECM-mediated immunosuppression in solid tumors.

## Structural composition and properties of the ECM

The ECM occupies the broad interstitial spaces of connective tissue as a hydrated, three-dimensional lattice. Rather than being a static scaffold, it is a dynamic composite in which fibrous collagens interacts with GAG chains, proteoglycan assemblies, and matricellular proteins. The relative abundance and molecular subtype of each building block are locally tuned, yielding microdomains that differ in terms of mechanical stiffness, porosity, and signaling capacity. The major ECM components and their functions are summarized in Table 1.

**Table 1:** Key ECM components and their functions

ECM components	Functions and Properties	Refs
Structural Proteins		
Collagens Formed as fibrils within the ECM (Collagen I, II, III, V and XI)	Provides mechanical strength, supports tissue architecture, regulates cell adhesion Promotes tumor cell migration, angiogenesis, modulates immune response via integrins, DDRs, and LAIR-1 interactions	[13–15]
Elastin	Maintains tissue elasticity and resilience Facilitates tumor plasticity, enhances metastatic potential	[16, 17]
FN	Supports cell adhesion, migration, and wound healing Modulates TME, promotes tumor invasion, interacts with α5β1 integrin to suppress immune infiltration	[18, 19]
Laminin	Maintains basement membrane integrity, regulates cell signaling Enhances tumor cell survival, promotes metastasis and chemoresistance	[8, 20, 21]
TNC	Involved in tissue remodeling and immune regulation Induces ECM remodeling, immune suppression, and metastatic niche formation	[22, 23]
Nonstructural Proteins & Proteoglycans		
HA	Regulates hydration, cell motility, and tissue repair Promotes tumor immune suppression via CD44, enhances M2 macrophage polarization	[24–26]
Versican	Modulates cell adhesion, migration, and inflammation Facilitates tumor progression, regulates cytokine signaling, and promotes metastatic niche formation	[27, 28]
Aggrecan	Provides structural and elastic support, maintains hydration and swelling, participates in tissue development and repair Transferrin (also known as the HA-binding complex) released by the Aggrecan core protein restricts the formation of tumor nodules in melanoma or Lewis lung cancer	[28, 29]
OPN	Regulates bone remodeling, immune cell migration Promotes tumor progression, immune cell recruitment, and therapy resistance via integrins	[30, 31]

*FN* Fibronectin, *TNC* Tenascin-C, *HA* Hyaluronan, *OPN* Osteopontin

The ECM constitutes a dense, highly insoluble network composed of proteins containing evolutionarily conserved structural domains. These domains exhibit significant conservation in their sequence and arrangement and are frequently subject to glycosylation. Many also bear sulfated glycosaminoglycan chains that impart a strong negative charge [32, 33]. This modular organization reflects conserved structure–function relationships, as exemplified by the dystroglycan-mediated recognition of specific integrin or non-integrin receptors, collagen domain-driven oligomerization, and calcium binding facilitated by C-type lectin domains. Nonetheless, the mosaic assembly of discrete domains can confer emergent functional properties, such as proteolytic cleavage and the subsequent release of isolated domains. The pronounced negative charge inherent to many ECM molecules, particularly proteoglycans, coupled with their expansive tissue distribution, enables extensive electrostatic interactions with charged ligands such as growth factors and chemokines. This modulates the local concentration and spatial distribution of these signaling molecules [32]. As a highly organized, insoluble suprastructure, the ECM is capable of spatially patterning and regulating the integration and delivery of complex signals that influence leukocyte behavior during inflammation.

## ECM remodeling in inflammation and cancer progression

ECM remodeling is a central biological process linking inflammation and cancer progression. In acute inflammation, the ECM acts as a dynamic signaling hub to regulate the initiation, amplification and extinction of immune responses [34]. When inflammation becomes chronic, dysregulation of the ECM and persistent inflammatory signaling form a vicious cycle, driving fibrosis and creating a microenvironment for tumorigenesis [33]. Ultimately, at the stage of tumor progression, the ECM undergoes profound changes in structure and composition, promoting tumor invasion, immune escape, and treatment resistance through remodeling of the basement membrane and interstitial matrix. This chapter will systematically elaborate the multiple roles and mechanisms of ECM in acute and chronic inflammation and cancer development.

### ECM remodeling in the acute inflammatory response

The ECM, once considered a static scaffold, has been redefined by single-cell sequencing and live imaging studies over the past five years as a dynamic signaling hub that actively participates in the initiation, amplification, and resolution of acute inflammation [34, 35]. Upon tissue injury, high-molecular-weight HA is cleaved by reactive oxygen species (ROS) and neutrophil-derived hyaluronidase into low-molecular-weight fragments (< 200 kDa). These fragments act through the toll-like receptor 2 (TLR2)/myeloid differentiation primary response 88 (MyD88) axis to stimulate resident macrophages to produce TNF-α and interleukin-1β (IL-1β), serving as an initial trigger of the cytokine storm [36, 37]. Circulating HA fragment levels correlate positively with neutrophil counts in the bronchoalveolar lavage fluid of patients with acute respiratory distress syndrome, the potential of HA fragments as liquid biomarkers for assessing acute inflammatory severity [37]. During the inflammatory initiation phase, activated vascular ECs enhance permeability, allowing plasma fibrinogen to extravasate and polymerize into fibrin, which forms a provisional ECM scaffold. This scaffold supports neutrophil adhesion and migration during subsequent phases. Through integrin α5β1 binding, it activates the focal adhesion kinase (FAK)/mitogen-activated protein kinase (MAPK) pathway in inflammatory cells, promoting the secretion of IL-8 and TNF-α from macrophages and thereby amplifying inflammation [38]. Concurrently, changes in ECM stiffness transduce mechanical signals via the HA/CD44/actin axis, thereby activating Piezo1 ion channels and the RhoA/ROCK pathway. This enhances pro-inflammatory polarization in macrophages and helps shift the response from inflammation toward repair [39]. Upon reaching the injury site, neutrophils release MMP-8 and MMP-9, which not only clear pathogens but also degrade collagen IV and laminin. This proteolysis generates RGD-motif-containing peptides that activate β₂ integrin-Rac1 signaling, prolonging neutrophil survival and enhancing ROS production, a positive feedback loop termed “ECM degradation-inflammation amplification” [40]. As inflammation resolves, ECM remodeling plays an active role in termination, and MMP activity is suppressed by tissue inhibitors of MMP (TIMPs). Under the influence of TGF-β and ED-A isoform FN, fibroblasts differentiate into myofibroblasts, which synthesize large amounts of collagen types I and III and FN. Thrombospondin-1 (TSP-1) promotes organized collagen crosslinking, whereas osteopontin (OPN) facilitates the transition of macrophages to the M2 phenotype. These M2 macrophages then secrete anti-inflammatory mediators such as IL-10 and TGF-β, thereby promoting ECM maturation and functional tissue recovery [41]. The disruption of this dynamic equilibrium can drive disease progression. In acute skin inflammation, excessive MMP activation leads to aberrant ECM degradation, whereas abnormal collagen cross-linking during the repair phase contributes to scar formation. In acute pancreatitis, the activation of pancreatic enzymes is accompanied by the upregulation of MMP-2 and MMP-9, and the resulting ECM degradation products further amplify the inflammatory response, therapy exacerbating pancreatic tissue necrosis [42, 43].

Thus, the ECM and acute inflammation form a functional network characterized by reciprocal causation and real-time regulation. The core mechanism lies in the three-dimensional interplay between ECM fragments, immune receptors, and mechanical signaling, offering a novel perspective for developing precise anti-inflammatory strategies through targeted ECM remodeling.

### ECM dysregulation in systemic chronic inflammation

A bidirectional regulatory relationship exists between chronic inflammation and ECM dysregulation. Under chronic inflammatory conditions, inflammatory cytokines and MMPs degrade ECM components, generating bioactive fragments that further modulate immune cell functions. In contrast, intact ECM molecules and their degradation products can directly affect immune cells and influence their activation, differentiation, and survival, thereby contributing to the pathogenesis of autoimmune diseases [33]. For instance, certain ECM fragments exhibit chemotactic activity or exacerbate inflammatory responses via pathways, such as TLR activation [44]. Persistent inflammatory signaling disrupts ECM homeostasis, promoting the sustained overexpression of MMPs by macrophages and fibroblasts. This degrades the ECM and generates proinflammatory fragments, establishing a vicious cycle [45]. TGF-β derived from M2 macrophages activates pro-fibrotic pathways, stimulating the synthesis and deposition of collagen types I and III as well as FN, thereby increasing ECM stiffness. Furthermore, the physical properties of the ECM-such as stiffness and architecture, can modulate inflammatory progression. In fibrotic tissues, excessive ECM deposition and cross-linking lead to tissue stiffening, which activates immune cells and promotes the release of inflammatory mediators [39]. Interactions between stromal and immune cells are critical for sustaining chronic inflammation. For example, in the lungs, innate immune cells regulate fibroblast behavior through cytokine secretion [46], which not only drives ECM remodeling but also helps maintain the local inflammatory milieu by secreting chemokines and cytokines.

The chronic inflammatory microenvironment resulting from ECM dysregulation significantly increases the risk of tumorigenesis. In mouse models of pancreatitis, tissue-resident macrophages (TRMs) exert protective effects by activating fibroblasts; however, the same mechanism promotes both fibrosis and tumor progression in pancreatic cancer [47]. Similarly, in breast and bladder cancers, elevated levels of HA fragments in the serum and TME accelerate tumor growth by promoting inflammation and angiogenesis [45].

### ECM in related chronic inflammation diseases

ECM dysregulation plays a central role in various organ-specific diseases. In the vascular system, the ECM constitutes the dynamic microenvironment of the vessel wall, and its abnormalities are closely associated with atherosclerosis, aortic aneurysms, and vascular aging. For instance, during atherosclerosis, increased degradation of collagen and elastin, coupled with elevated MMP activity, promotes plaque development and vascular remodeling [48]. Organ fibrosis is a classic manifestation of ECM dysregulation and is characterized by excessive ECM deposition and the disruption of tissue architecture. Abnormal proliferation and activation of lung fibroblasts have been observed in idiopathic pulmonary fibrosis (IPF). GDF15, a key regulatory factor and member of the TGF-β family, is upregulated in response to tissue injury or inflammation, enhancing the fibrotic response of fibroblasts and driving disease progression [49].

The progression of liver fibrosis is characterized by a significant increase in the synthesis and deposition of ECM proteins. The content of collagen type I can reach up to eight times that of a healthy liver, while collagen type III, fibronectin, and laminin also accumulate in the space of Disse, forming a dense matrix [50]. Without intervention, some fibrosis patients may progress to cirrhosis or hepatocellular carcinoma (HCC), with the risk of progression closely linked to the underlying etiology [51]. Although the reasons for these differences are not yet fully understood, it is noteworthy that both the stage of fibrosis and its etiology appear to be associated with structural and compositional changes in the ECM. For example, during chronic hepatitis C (HCV) infection, ECM components undergo stage-specific evolution, marked by the gradual accumulation of type I and III collagen, followed by the overexpression of elastin [52]. Chronic infections with HBV and HCV drive inflammatory and antiviral suppressive immune responses, often leading to progressive fibrosis and ECM remodeling [53]. Daneshgar et al. observed that in decellularized human liver samples, the expression of MMP 23B, MMP 28, and versican increased with advancing fibrosis and cirrhosis [54].

Furthermore, chronic inflammatory skin diseases are often characterized by the ECM being trapped in a feedback loop, exacerbating the disruption of homeostasis and leading to chronic alterations in ECM degradation. This results in the accumulation or loss of certain barrier components and a non-healing phenotype [55]. For instance, lesions of hidradenitis suppurativa (HS) show high gene expression of MMP-1, −2, −3, −9, and −10, which correlates with IL-1β protein levels [56, 57]. Overexpression of MMP-2 has been detected in inflammatory cells within keratinocytes, fibroblasts, and the dermis—including sweat glands, hair follicles, and sinus tracts from HS-affected skin [58]. MMP-induced alterations may facilitate the release of bioactive peptides and inflammatory factors, thereby promoting HS pathogenesis [56]. Psoriasis is a common chronic inflammatory skin disease affecting approximately 3% of the global population. Its pathogenesis involves a combination of genetic, environmental, and immune factors, particularly driven by type 1 and type 17 T helper cells [59]. Studies have highlighted the role of syndecan-1 in regulating Tγδ17 cell homeostasis and modulating psoriasiform skin inflammation [60]. These immune disturbances lead to hallmark features of psoriasis, including hyperproliferation and abnormal differentiation of keratinocytes, altered angiogenesis, and changes in ECM components such as laminin and fibronectin [61]. Cytokines regulate MMP production, which is involved in the pathological processes of psoriasis and crucial for the degradation of the ECM and basement membrane [62].

ECM dysregulation is a central pathological feature of various chronic inflammatory diseases. In organs such as the vasculature, lungs, liver, and skin, the disruption of ECM homeostasis—manifested as excessive degradation or abnormal deposition—forms a vicious cycle with sustained inflammatory responses, jointly driving disease progression. The ECM serves not only as a structural scaffold but also as a critical source of signals that regulate inflammation and repair. Future research should focus on elucidating how specific ECM components or degradation products (e. g., matrikines) precisely modulate immune cell function. This will provide key targets for developing novel therapeutic strategies aimed at the ECM-inflammation axis.

### The association between chronic inflammation and cancer

Pro-carcinogenic inflammation is often initiated and sustained even before the onset of tumor formation [63]. Inflammatory bowel disease, chronic viral hepatitis, Helicobacter pylori-associated gastritis, and schistosomal cystitis significantly increase the risk of colorectal cancer, HCC, gastric cancer(GC), and urothelial carcinoma, respectively [64]. It is widely accepted that chronic inflammation serves as a key carcinogenic trigger by driving the accumulation of mutational burden in healthy cells [65]. Neutrophils and macrophages constitute the primary cellular sources of ROS and reactive nitrogen intermediates, whose release induces oxidative/nitrosative stress that accelerates mutation accumulation in normal tissues [66]. For instance, persistent intestinal inflammation is closely associated with the gradual enrichment of driver gene mutations such as *TP53* in intestinal epithelial cells [67]. Moreover, several inflammatory factors directly contribute to mutagenesis: IL-22 upregulates DNA damage repair genes to counteract inflammation-associated genotoxicity [68]. TNF-α and IL-1 remodel the transcriptional activity of oncogenes and tumor suppressor genes by activating epigenetic regulators such as Dnmt1 and DOT1L, as well as modulating miRNA and lncRNA networks—effects that closely resemble the consequences of mutations that inactivate tumor suppressors or activate oncogenes [69]. A third link between chronic inflammation and tumor initiation lies in the reprogramming of stem cell fate: inflammatory signals can induce quiescent epithelial cells to dedifferentiate into tumor-initiating stem cells. This process is accompanied by impaired epithelial barrier integrity, exposing the stem cell niche directly to environmental carcinogens or genotoxic metabolites produced at inflammatory sites [70]. This mechanism is particularly significant, as these "reprogrammed" stem cells often serve as "seeds" for metastatic dissemination, driving the development of tumors in secondary organs.

### ECM structural changes during tumor progression

**Fig. 1:**
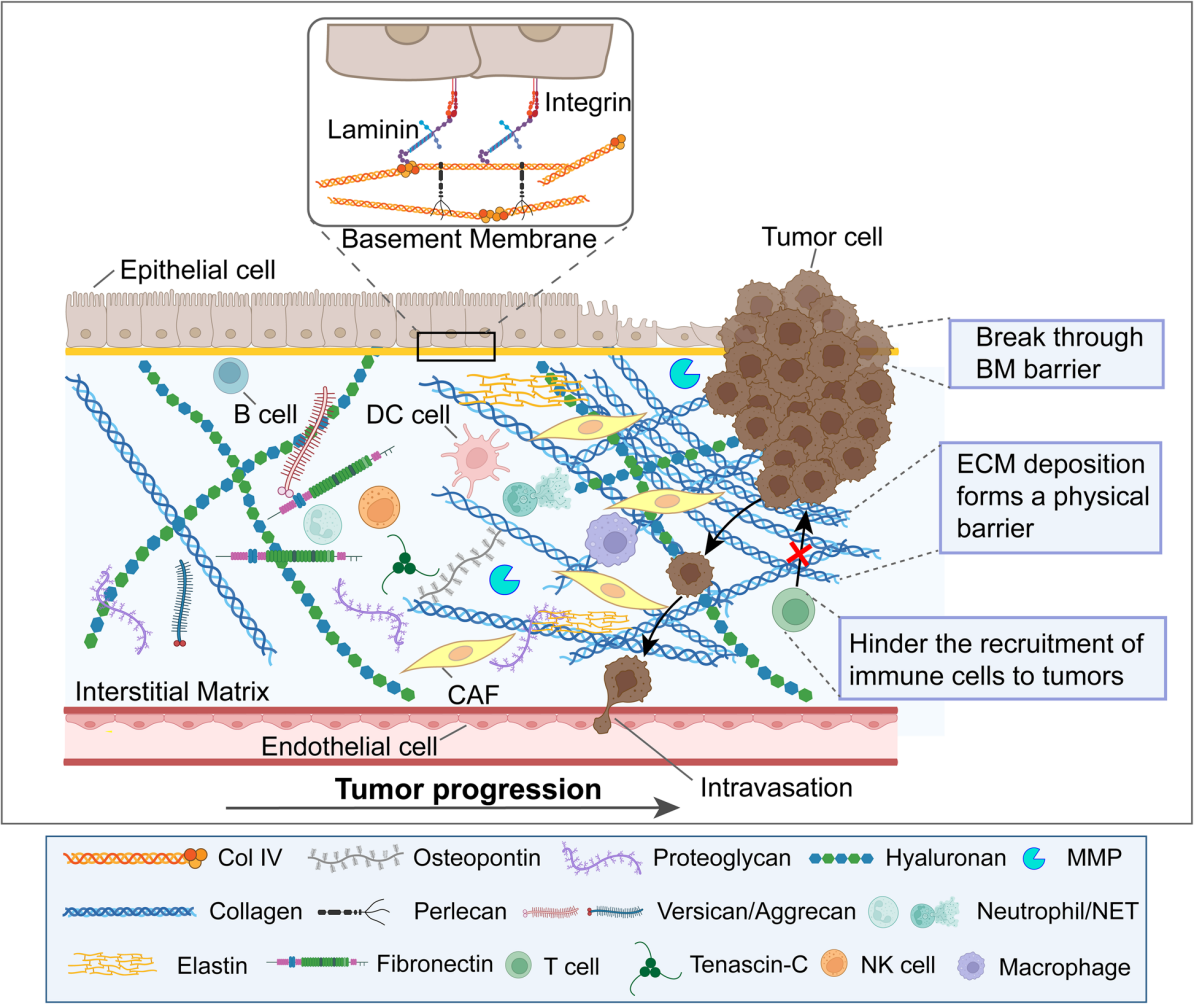
The composition and structural changes of the TME during cancer progression and the key representative cell types are shown. The TME includes CAFs, immune cells, endothelial cells, and the ECM. As cancer progresses, cancer cells break through the structural barrier of the ECM basement membrane, leading to increased proliferation, invasion, and endocytosis at the primary site; deposition of interstitial matrix; and increased ECM stiffness, forming a physical barrier in the TME, thereby affecting ECM-cell interactions, impeding recruitment of immune cells to the tumor, leading to immune rejection, and modulating immune cell activity in tumor progression leading to immunosuppression. BM: Basement membrane; DC: Dendritic cell; ECM: Extracellular matrix; CAF:Cancer-associated fibroblast; CoI IV:collagen type IV; MMP: Matrix metalloproteinase

At the structural level, the ECM is divided into the basement membrane (BM) and the interstitial matrix (IM). The BM supports the epithelium and ECs, whereas the IM supports a broader interstitial compartment. Degradation of the peripheral ECM is a critical factor in tissue destruction and a key component of invasive tumor growth [71]. As the tumor progresses, ECM remodeling is accompanied by the deposition of tumor-specific ECM, which typically increases in density and stiffness [72] (Fig. 1).

#### Basement membrane

The BM is a specialized, dense ECM structure that primarily consists of type IV collagen, laminin, FN, perlecan, and nidogen. It serves as a critical barrier that separates endothelial and epithelial cell layers from the interstitial ECM, maintaining tissue architecture and regulating cell behavior [73]. However, in the TME, the BM undergoes significant remodeling, which plays a pivotal role in cancer progression, immune evasion, and therapeutic resistance.

During cancer initiation and progression, BM degradation facilitates tumor cell invasion, extravasation, and intravasation. Cancer cells disrupt BM integrity either by exploiting preexisting openings or by secreting ECM-remodeling enzymes, particularly MMPs and cathepsins, which cleave BM components to facilitate migration [10, 74]. MMP-2 and MMP-9, for example, specifically degrade type IV collagen, weaken the BM structure, and promote invasion [75]. Epithelial-mesenchymal transition (EMT) also contributes to BM remodeling, as cancer cells undergoing EMT secrete additional proteases and downregulate adhesion molecules such as E-cadherin, further weakening cell-BM interactions [76].

In more advanced tumors, the BM undergoes excessive thickening rather than degradation. This is characterized by the lamellar accumulation of collagens I, III, IV, FN, and laminin, creating a physical barrier that segregates tumor nests from the stromal components [75–77]. Excessive BM deposition is particularly evident in aggressive tumor types where it enhances mechanical stiffness, limits immune cell infiltration, and contributes to therapy resistance [78, 79]. For instance, in pancreatic ductal adenocarcinoma (PDAC), thickened BM restricts cytotoxic T-cell infiltration, impairs immune surveillance, and promotes an immunosuppressive TME [80].

Beyond its role in structural integrity, the BM functions as a reservoir for bioactive molecules, including growth factors (TGF-β, vascular endothelial growth factor[VEGF], epidermal growth factor[EGF]) and cytokines, which influence tumor growth, immune cell recruitment, and angiogenesis [81]. Laminin, for example, interacts with integrin receptors (e. g., α6β4 and α3β1) on cancer and immune cells, activating signaling pathways that enhance tumor cell survival, migration, and immune evasion [82]. Additionally, type IV collagen-derived fragments (e. g., tumstatin and canstatin) can exert either pro-or anti-tumor effects depending on their interactions with the immune system and ECs [83].

Given the dynamic interplay between BM remodeling and tumor progression, targeting BM-associated pathways is a promising therapeutic strategy. Inhibiting MMP activity, modulating integrin signaling, or disrupting tumor-BM adhesion may help restore BM integrity, enhance immune infiltration, and improve therapeutic efficacy [84]. Future research should focus on understanding the heterogeneity of BM remodeling across different cancer types and its implications in immunotherapy resistance.

#### Interstitial matrix

The IM includes glycoproteins, such as collagen, elastin, proteoglycans, HA, laminins, and FN [78] that embed CAFs, immune cells, blood vessels, and the lymphatic vasculature [3]. Increased fibrillar collagen deposition in cancer and denser collagen fibers in the IM are associated with high and organized accumulation [75, 81, 82]. As the tumor progresses, the arrangement of interstitial collagen fibers near the BM increases, particularly at the edge of the tumor, facilitating tumor invasion [83–85]. Overexpression of ECM glycoproteins such as tenascin-C (TNC), which is associated with severe tumor progression in various cancers [86–88], supports a stem cell phenotype in the metastatic niche [89]. Versican, a large chondroitin sulfate-containing proteoglycan, can bind HA with high affinity, forming distinct HA-rich matrices that commonly accumulate during inflammatory processes [90]. These ECM glycoproteins that mediate tissue inflammation are overexpressed in cancer cells and form an ecological niche that promotes the migration, adhesion, and metastasis of cancer cells [91].

## Molecular mediators and immune cells involved in ECM remodeling in inflammation and cancer

ECM remodeling is a dynamic process that is precisely regulated by a variety of molecular mediators and immune cells. In this network, MMPs, disintegrin and metalloproteinases (ADAMs) and lysyl oxidases (LOXs) are the core protease systems responsible for the degradation and cross-linking of ECM [92–94]. Cytokines, chemokines, and hypoxia-inducible factor (HIF) profoundful regulate the structure and function of the ECM at both biochemical and physical microenvironment levels [95–97]. As major producers of ECM, stromal cells, represented by CAFs, exhibit remarkable plasticity [98]. At the same time, bone marrow-derived immune cells such as neutrophils, macrophages and various types of lymphocytes not only secrete remodeling enzymes, but also have their functions regulated by ECM degradation products, forming a complex bidirectional interaction network [99]. In this chapter, We will elaborate on how these key mediators and cells jointly drive ECM remodeling in inflammation and cancer.

### MMPs, ADAMs, and LOXs

Given the crucial role of MMPs in normal physiology, their dysregulation has been implicated in severe pathological disorders, including cancer and inflammatory diseases [80, 100]. MMPs, a family of zinc-dependent endopeptidases, are critical mediators of ECM remodeling, capable of facilitating tissue homeostasis as well as driving pathological processes, such as tumor progression. Although CAFs are a major source of MMPs, other stromal and immune cells, including tumor-associated macrophages (TAMs) and tumor-associated neutrophils (TANs), also contribute to MMP-mediated ECM degradation, amplifying their impact on the TME [92]. MMPs are classified as gelatinases (MMP-2, −9), collagenases (MMP-1, −8, and −13), stromelysins (MMP-3, −10, and-11), and membrane-type MMPs (MT-MMPs, e. g., MMP-14), each exhibiting substrate specificity that determines their role in ECM remodeling [101–103]. Among the 30 known MMPs, MMP-2, −3, −9, and −14 have been implicated in tumor progression. These enzymes degrade type IV collagen, FN, and laminin, facilitating BM breakdown, tumor invasion, and immune cell trafficking [104]. The spatial localization and proteolytic activity of MMPs in the TME are critical determinants of cancer progression. For example, elevated MMP14 expression in the cell membrane enhances the invasive capacity of epithelial carcinoma cells through invadopodia-specialized actin-rich protrusions that are highly enriched in MMPs and facilitate ECM degradation [105]. Beyond degradation, MMP activity is tightly regulated by TIMPs, which modulate the ECM remodeling dynamics. An imbalance between MMPs and TIMPs not only promotes ECM remodeling but also alters immune infiltration, affecting the efficacy of immunotherapies [106]. For instance, MMP-9 enhances immune suppression by modulating TGF-β activation, whereas MMP-14 facilitates ECM stiffening, creating a physical barrier that impairs T-cell infiltration [107]. With an in-depth study of intercellular interactions, the role of the ECM and tissue integrity in infection biology has gradually emerged. During infection, the protection of microorganisms by the body's epithelial barriers (such as the skin, lungs, and intestines), supported by ECM scaffolds, is severely hindered and destroyed [108]. MMP-mediated immune-regulatory ECM remodeling, while promoting the infiltration of immune cells into the infection site, may also lead to severe and irreversible pathological damage [109]. An infection with specific pathogens can disrupt ECM homeostasis, and the upregulation of cytokine levels during inflammation can further stimulate the production of proteolytic enzymes such as MMPs by monocytes [110, 111]. In this context, MMP-3 and MMP-9 have been identified as potential biomarkers of COVID-19 severity [112]. In a study of mouse models infected with SARS-CoV-2, increased expression of MMP-14, MMP-8, and MMP-9 was observed in the lung tissue after infection. These changes were closely related to ECM degradation and subsequent tissue damage [113, 114].

Notably, the ADAM family of proteases also plays a pivotal role in cancer and inflammatory processes. Cancer cells frequently exhibit elevated ADAM expression, which contributes to enhanced cell adhesion and proteolytic activity, thereby facilitating tumor progression within the TME [93]. Illustrative examples include the proteolytic shedding of the inflammatory cytokine TNF-α by ADAM17 [115, 116], as well as the cleavage of CD23, a receptor involved in modulating immune responses, by ADAM10 [117]. ADAM proteins contribute significantly to inflammation-related tissue damage and disease progression by modulating immune cell function and migration, tissue injury, ulcer formation, and ECM deposition and degradation. For instance, ADAM17 facilitates trans-signaling cascades through the shedding of the IL-6 receptor and processing of EGF ligands, thereby playing a key role in inflammatory and cancerous pathologies [115, 118].

The LOX family, which includes LOX and LOX-like proteins 1–4 (LOXL1-4), catalyzes the covalent crosslinking of collagen and elastin. This activity is essential for ECM stability and supports key biological processes, including connective tissue integrity, embryonic development, and wound repair [94]. Collagen cross-linking is a critical step in collagen synthesis, which organizes the precollagen chains into a triple helical structure of collagen. This process promotes the oxidative deamination of lysine and hydroxylysine by the LOX family members to form reactive aldehydes. These aldehydes spontaneously form crosslinks with neighboring lysine or hydroxylysine residues, thereby enhancing the ECM stiffness [119]. LOX-mediated collagen cross-linking increases ECM stiffness by 3–fivefold in PDAC [120]. These enzymes are key catalysts in ECM remodeling, covalently cross-linking collagen and elastin to enhance ECM stiffness and mechanical stability. In the TME, tumor-initiating cells secrete LOX, which promotes collagen cross-linking and initiates a signaling cascade involving upregulation of integrin α7, activation of FAK/Src, and phosphorylation of extracellular signal-regulated kinase 1/2 (ERK1/2). In addition to tumor cells, CAFs can directly mediate collagen cross-linking by secreting small extracellular vesicles (sEVs) enriched with LOX. The underlying mechanism involves the release of αLOX-rich sEVs by CAFs that recognize collagen I via the surface receptor integrin α2β1, thereby initiating collagen cross-linking. This crosslinked collagen matrix subsequently promotes EMT in oral squamous cell carcinoma (OSCC) by activating the p-FAK/p-paxillin/YAP signaling pathway [121].

Moreover, YAP1-positive CAFs can upregulate the expression of stiffness-related genes such as LOX in liver cancer, promoting ECM remodeling and increasing tumor tissue rigidity, thereby creating favorable conditions for tumor cell survival and proliferation [122]. Similarly, in salivary adenoid cystic carcinoma, LOX secreted by CAFs enters the systemic circulation, induces YAP expression and its nuclear translocation, and activates metastasis-associated fibroblasts (MAFs) in the lung. This enhances type I collagen expression in MAFs, promoting collagen cross-linking within the pulmonary pre-metastatic niche and facilitating lung metastasis [123]. In cholangiocarcinoma (CCA), inflammatory cancer-associated fibroblasts (iCAFs)secrete substantial amounts of LOX, which is internalized by tumor cells. Through protein–protein interaction with mitochondrial transcription factor A, LOX reprograms CCA metabolism. By improving metabolic fitness, modulating mitochondrial function, and enhancing stem-like properties, LOX supports tumor initiation and progression both in vitro and in vivo [124]. In GC, LOX derived from CAFs in the liver metastatic niche promotes GC tumor cell proliferation by enhancing the AKT-p70S6K-HIF1-α pathway-mediated Warburg effect. Concurrently, tumor cells in the GC microenvironment secrete TGF-β1, which in turn stimulates CAFs to produce more LOX, forming a vicious cycle that drives tumor progression [125]. This sequence of molecular events increases ECM stiffness, alters tumor-stroma interactions, and contributes to cancer progression and therapeutic resistance [106, 107].

MMP and LOX play crucial roles in ECM remodeling and neosynthesis. While LOX facilitates ECM assembly, MMPs degrade the ECM components. These proteins exhibit antagonistic biological functions. However, studies have revealed a positive correlation between LOX and MMP-2 expression in GC tissues, where they act synergistically to promote tumor invasion and metastasis [126]. In GC models, the LOX inhibitor β-aminopropionitrile (BAPN) significantly reduced the expression and activity of MMP-2/9, whereas exogenous LOX increased MMP-2/9 levels in a dose-dependent manner. Mechanistically, LOX oxidatively activates platelet-derived growth factor receptor α/β (PDGFR-α/β), enhancing platelet-derived growth factor (PDGF) signaling, which subsequently upregulates MMP-2/9 transcription. This establishes a sequential "cross-linking-degradation" process that collectively drives tumor invasion [127]. In a mouse model of thyroid cancer metastasis, nuclear-localized LOX was recruited to the Slug (SNAI2) promoter and transcriptionally upregulated SNAI2. SNAI2 promoted the secretion of TIMP4, which inhibited MMP-2/9 activity, thereby confining the "degradation pulse" to the LOX-high invasion front. This mechanism achieves a fine-tuned balance between ECM cross-linking and proteolysis [128]. Furthermore, MMP and LOX exhibit intriguing relationships with TGF-β. Under various inflammatory pathologies, TGF-β is upregulated and induces the expression of LOX family enzymes as well as various MMPs [129, 130]. In addition to inducing the expression of MMPs, TGF-β can also modulate TIMP expression, highlighting the complexity of inflammatory pathology [131].

### Cytokine and chemokine modulation of ECM architecture

The ECM serves as a rich reservoir of chemokines and cytokines, the bioavailability of which is regulated by the capacity of immune cells to access these components. The bioavailability of TGF-β, one of the most important cytokines in ECM regulation, is tightly controlled by its regulated release from the ECM [53]. Most hepatocytes are sensitive to TGF-β1. During fibrogenesis, excessive TGF-β1 activates hepatic stellate cells, inducing their transdifferentiation into myofibroblasts, which, in turn, deposit more ECM proteins. TGF-β1 also promotes ECM deposition by amplifying hepatocyte cell death [132]. Furthermore, during liver fibrosis, the timely release of TGF-β1 in response to changes in ECM stiffness can drive both pro-inflammatory and immunosuppressive responses. For instance, TGF-β1 is a key mediator of the terminal differentiation of regulatory T cells (Tregs), which act as critical negative regulators of inflammatory processes in liver fibrosis [133]. The mechanical state of ECM fibers, such as their capacity to stretch or unfold, can also influence cytokine availability or activity [134]. For example, a large reservoir of latent TGF-β complexes stored within the ECM can be activated by mechanical forces, and stiffer matrices lower the activation threshold for TGF-β1 [135].

In addition, type 2 cytokines, particularly IL-13, have emerged as regulators of ECM quantity and quality, including modulation of the mucosal barrier [96]. During defense against gastrointestinal nematode infections, IL-13-induced goblet cell hyperplasia is essential; the mucus produced in this process is essentially a carbohydrate-rich gel-like ECM structure [136]. Furthermore, IL-13 not only increases the mucus volume but also alters its composition. It specifically induces Muc5AC mucin, whose overproduction is a hallmark of allergic airway inflammation and is necessary for nematode expulsion [95, 137].

Although cytokines such as TGF-β and IL-13 regulate the ECM by driving its production or enzymatically modulating its composition, direct biophysical interactions between the ECM and chemokines or cytokines can alter ECM structure or function. For instance, CXCL4 (PF4) functions by binding to GAGs, rather than directly to chemokine receptors. This interaction may remodel the cell surface-associated ECM and influence signaling through proteoglycans [138]. Such effects could be mediated via signaling through cell surface proteoglycans and/or remodeling of the glycocalyx to promote leukocyte-endothelial interactions [139]. Moreover, ECM proteins bind chemokines to form chemotactic gradients that recruit and activate immune cells. For example, in OSCC, TNC induces and binds to C–C motif chemokine ligand 21 (CCL21) to establish an immunosuppressive lymphostromal niche, recruit Tregs, and promote anti-inflammatory cytokine expression, thereby exacerbating the immunosuppressive microenvironment [140].

### Hypoxia and HIF signaling in ECM remodeling

As tumors progress, the formation of a hypoxic microenvironment activates HIF-1-dependent signaling pathways, enabling both tumor and stromal cells to adapt to low-oxygen conditions and further promote tumor development [97]. HIF-1 contributes to ECM remodeling by upregulating the expression of various MMPs, such as MMP-2, MMP-9, and MT1-MMP [141, 142]. Additionally, HIF-1 induces the expression of multiple collagen-modifying enzymes, including prolyl 4-hydroxylases (P4HA1 and P4HA2), PLOD1, PLOD2, LOX, LOXL1, LOXL2, and LOXL4 in CAFs-thereby promoting the organized assembly of collagen fibers and facilitating tumor progression [143–145]. Hypoxia is a key driver of tumor angiogenesis. Under low oxygen conditions, tumor cells upregulate and activate critical factors such as VEGF via HIF-1, recruiting ECs and initiating the "angiogenic switch", which sets the stage for new blood vessel formation [146]. Endothelial tip cells must remodel the surrounding ECM for these nascent vessels to function. This process involves the upregulation of collagen-modifying enzymes, including members of the LOX and PLOD families, a mechanism confirmed in multiple human cancers [147]. Hypoxia and neutrophil infiltration are commonly observed in muscle-invasive bladder cancer and are associated with resistance to immunotherapy. Hypoxia-driven ECM remodeling can modulate neutrophil recruitment, polarization, and activation through biomechanical and biochemical signaling, influencing the polarization of neutrophils toward either pro-tumor or anti-tumor phenotypes. Such alterations in ECM under hypoxic conditions can affect the efficacy of T cell-based immunotherapies [148].

### Fibroblast and myofibroblast plasticity in ECM production

Fibroblasts serve as the primary source of ECM components under both physiological and pathological conditions. Upon activation, these cells differentiate into myofibroblasts, acquiring a hybrid phenotype that combines features of fibroblasts and smooth muscle cells. In addition to producing and secreting ECM constituents, myofibroblasts possess contractile capabilities, thereby modulating the mechanical properties of the ECM in a three-dimensional context. The activation of myofibroblasts is regulated by various pro-inflammatory factors, with TGF-β playing a pivotal role [149]. Maintaining a dynamic balance between activation and deactivation is essential for preserving ECM homeostasis and ensuring proper tissue repair, particularly during wound healing and regeneration. However, under pathological conditions, persistent inflammatory stimuli and sustained TGF-β release from immune and tumor cells disrupt this balance, leading to excessive proliferation and activation of myofibroblasts. This, in turn, contributes to the development of fibrotic diseases and the characteristic dysregulated stromal remodeling observed in the TME [150, 151].

CAFs are primarily derived from tissue-resident or bone marrow-derived fibroblasts, although they may also originate from mesenchymal stem cells and other cell types undergoing epithelial-mesenchymal transition [98, 152]. CAFs are one of the most abundant stromal cell types in the TME and remain the main force involved in ECM deposition and remodeling [153, 154]. Numerous studies have underscored the pivotal role of CAFs in ECM remodeling [155–157]. Although CAF subtypes exhibit functional heterogeneity, with some promoting tumor progression and immunosuppression and others exhibiting tumor-restraining functions, a consistent finding is their central role in driving tumor progression and fibrosis [158, 159]. Recent advances in single-cell technology have revealed previously unrecognized CAF subtypes and their functional diversities [3]. For example, two CAF-S1 clusters in breast cancer (BC) were identified through scRNA-seq, namely ECM-myCAFs and TGF-β-myCAFs. ECM-myCAFs stimulate programmed death 1 (PD-1) and cytotoxic T-lymphocyte-associated protein 4 (CTLA-4) protein expression in CD4^+^ CD25^+^T lymphocytes, creating an immunosuppressive environment that is crucial for immunotherapy resistance [160]. CAFs serve as primary drivers of collagen fiber synthesis and deposition in the ECM [161]. Distinct collagen expression profiles exist among different CAF subtypes; for instance, myoCAFs specifically upregulate COL10A1 and COL11A1, whereas iCAFs predominantly express COL14A1 [162] further highlighting this heterogeneity. Lambrechts et al. identified five CAF subpopulations in non-small cell lung cancer (NSCLC) using scRNA-seq. Among these, cluster 1 exhibited high expression of COL10A1 and was enriched in ECM-related proteins and TGF-β signaling genes. In contrast, cluster 2 showed elevated expression of COL4A1 along with marked upregulation of ACTA2, MEF2C, and MYH11 [163]. Owing to their central role in ECM deposition, CAFs contribute to the formation of a physical barrier that impedes the infiltration of immune cells and therapeutic agents into tumor tissues. This mechanism aids tumor cells in evading immune-mediated clearance and confers resistance to anticancer treatments [164]. Preclinical research suggests that CAFs inhibit the recruitment and activation of T cells either by releasing CXCL12 and TGF-β or by creating physical obstacles through ECM deposition [165, 166]. Moreover, CAFs enhance the degradation of normal ECM structures and increase matrix stiffness by secreting various matrix proteins such as FN and type I collagen (COL I), and generating diverse MMPs such as MMP1 and MMP3 [167]. PDGFRα^+^ITGA11^+^CAFs secrete chitinase 3-like protein 1 (CHI3L1) to upregulate the expression level of MMP2 and mediate remodeling of the ECM, which is very important for vascular invasion and lymph node metastasis in bladder cancer [168].

### Bone marrow-derived immune cells drive ECM remodeling

Bone marrow-derived cells, such as TANs and TAMs, serve as a significant source of ECM-remodeling proteases in the TME and metastatic sites [99].

TANs are key players in the tumor inflammatory microenvironment and release numerous bioactive proteases that facilitate tumor cell proliferation, invasion, metastasis, and angiogenesis [169]. MMPs are the most studied and prominent protease family related to tumorigenesis. MMP9 degrades components of the ECM and BM, contributes to tumor progression, and is essential for tumor angiogenesis and metastasis [170]. TGF-β in the TME can induce TAN polarization to the N2 type, producing significant amounts of MMP9 and VEGF, which are crucial for tumor angiogenesis [171, 172].

Activated neutrophils generate neutrophil extracellular traps (NETs) composed of decondensed nuclear or mitochondrial DNA along with histones, proteases, and other inflammatory mediators [173]. Recently, the involvement of NETs in tumor progression has garnered significant attention within the research community. Investigations into the relationship between NETs and tumor advancement have primarily focused on neutrophil-derived proteins such as neutrophil elastase (NE) and MMP9. These proteins facilitate tumor growth and migration by degrading the ECM [169]. The effect of NETs on tumors is indirectly mediated by ECM remodeling. The continuous inflammatory processes of neutrophils in the lungs form NETs. NET proteases, NE and MMP9, cleave laminin. This proteolytic remodeling culminates in the unveiling of distinct laminin epitopes, promoting integrin α3β1 signaling activation and subsequently fostering the proliferation of cancer cells [174].

MMPs secreted by TAMs are crucial mediators of cancer metastasis. Specifically, TAMs exhibiting elevated levels of B7-H3 in triple-negative breast cancer (TNBC) release MMP-2, TGF-β, and VEGF-A, orchestrating a microenvironment conducive to metastasis. These regulatory mediators hasten the breakdown of the ECM and neovascularization. A key mechanism driving tumor metastasis is the remodeling of the ECM by TAMs. TAMs that overexpress MMP11 promote tumor invasion and metastasis by activating the MAPK signaling pathway via the CCL2/CCR2 axis, leading to upregulation of MMP9. This cascade enhances ECM degradation, thereby facilitating migration and invasion of Human epidermal growth factor receptor 2 (HER2)^+^ breast carcinoma cells [175, 176]. These findings highlight the distinct and context-dependent roles of TAMs in various cancer subtypes, emphasizing the need for targeted therapeutic strategies to modulate TAM-mediated ECM remodeling, which contributes to ECM deposition in addition to the production of ECM-remodeling enzymes that degrade the ECM. TAMs direct the deposition, cross-linking, and linearization of collagen fibers in invasive areas of the tumor. In contrast, the lack of TAMs significantly reduces the crosslinking and density of collagen and notably attenuates the expression of collagen types I and XIV within CAFs [177]. Interestingly, TAMs can also influence collagen fiber orientation and pre-metastatic microenvironment formation by secreting oncostatin M (OSM), an inducer of LOXL2 expression in PDAC [178].

### Role of immune cells in ECM remodeling

**Fig. 2:**
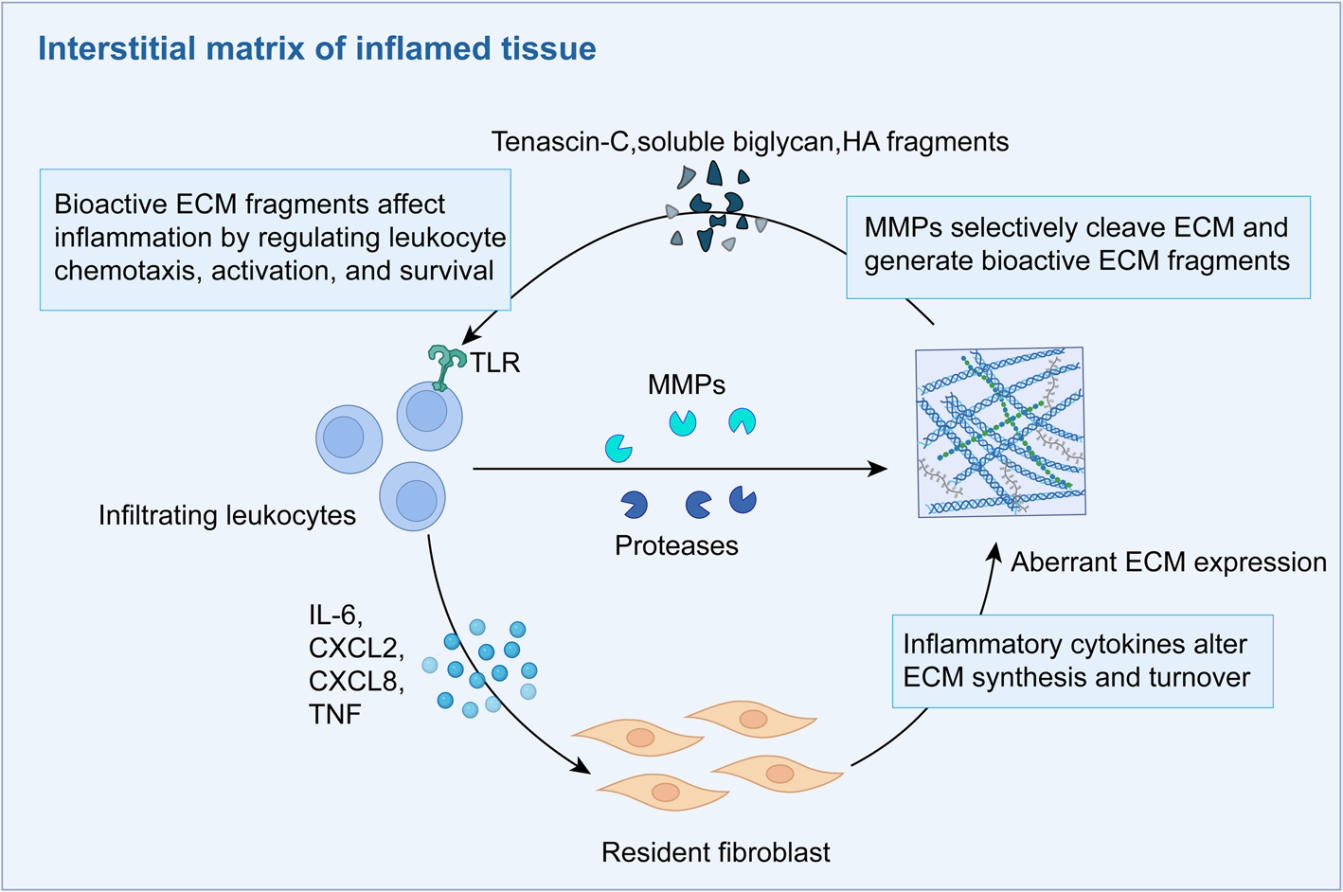
The intrinsic mechanism of ECM-mediated immune cell activation. During the process of chronic inflammation, infiltrating immune cells activate resident cells in tissues by secreting various cytokines and MMPs, and cause changes in ECM synthesis or selective lysis of their specific domains. Abnormal ECM expression and the biologically active ECM fragments it generates can influence the inflammatory process by regulating the chemotactic, activation and survival of immune cells, and can also promote the persistence of inflammatory responses by activating TLR2/TLR4. The above-mentioned mechanism usually occurs in the interstitial tissue under chronic inflammatory conditions. HA: Hyaluronan; ECM: Extracellular matrix; MMPs: Matrix metalloproteinases; TLR:Toll-like receptor; IL-6: Interleukin-6; CXCL2: Chemokine (C-X-C motif) ligand 2; CXCL8: Chemokine (C-X-C motif) ligand 8; TNF: Tumor necrosis factor

In inflamed tissues, the turnover of ECM and the secretion of proteases by tissue-resident cells are modulated by cytokines released from infiltrating immune cells, such as TGF-β, TNF-α, and IFN-γ. Accumulating evidence has indicated that abnormally expressed ECM components can influence immune cell activation, differentiation, and survival. Some of these molecules, particularly MMP-generating bioactive peptides that function as chemoattractants or modulators of immune cell activity, are selectively cleaved by proteases. Consequently, ECM remodeling in inflammatory microenvironments contributes to the propagation and chronicity of inflammation [33] (Fig. 2).

To date, the most compelling in vivo evidence for the generation of bioactive ECM fragments through selective proteolysis derives from studies on collagen I-derived chemotactic fragments. In the rheumatoid arthritis (RA) synovium, TNF-α and IL-17 induce fibroblast-like synoviocytes to upregulate MMP-1, MMP-3, and MMP-13. These enzymes cleave collagen I to release N-terminal tripeptides containing a Pro-Gly-Pro (PGP) motif. These PGP fragments mimic the function of CXCL8 by binding to CXCR1/2 receptors and promoting neutrophil recruitment, thereby establishing a positive feedback loop involving inflammation, collagen degradation, and neutrophil infiltration. Such PGP fragments have been detected in the joint fluid of patients with RA and may serve as biomarkers of disease activity [179]. Similarly, cleavage of laminin-5 in the BM by MMP-9 releases a peptide (ASKVKV) derived from the β4 chain. This peptide induces TNF-α secretion by monocytes and upregulates MMP-9 expression in vitro, suggesting that ECM fragments not only exert chemotactic effects but may also amplify tissue destruction by enhancing protease cascades [180]. The chemotactic activity of the ECM fragments is mediated by various cell surface receptors. For instance, the 67-kDa protein, also known as the high-affinity laminin receptor expressed on neutrophils, recognizes fragments derived from type IV collagen, laminin, and elastin, facilitating direct cell migration [181]. Notably, a single ECM component may be recognized by multiple inflammation-related receptors, and its proteolytic cleavage can yield fragments with distinct or even opposing biological functions. For example, neutrophils express several type IV collagen-binding receptors, including elastin-binding protein (EBP) and L-selectin [182]. Although peptides binding to the 7S domain of type IV collagen exert chemotactic effects via the EBP complex [183], another peptide derived from the α3 chain of type IV collagen has been reported to inhibit neutrophil activation [184]. Moreover, elastin degradation products, particularly those generated by macrophage elastase MMP-12, exhibit chemotactic activity for monocytes in chronic pulmonary inflammation [185].

In addition to their chemotactic roles, ECM-derived fragments and altered matrix molecules can directly activate immune cells and promote inflammatory responses [53]. Recent studies have shown that elastin fragments activate T cells to secrete IL-17, induce oxidative stress, and promote monocyte migration, thereby promoting the progression of inflammatory diseases such as atherosclerosis [45]. TLRs recognize conserved pathogen-associated molecular patterns (PAMPs) and trigger innate immune responses that shape adaptive immunity [186, 187]. Certain ECM components or fragments act as endogenous ligands for TLRs, particularly TLR4 and TLR2. In a murine model of RA, upregulation of TNC stimulated TLR4 in resident fibroblasts and macrophages, inducing the production of pro-inflammatory cytokines such as IL-6, TNF-α, and CXCL8. Mice deficient in TNC were protected from zymosan (a TLR2 agonist)-induced synovitis, suggesting that TNC helps sustain joint inflammation and propagates local inflammatory responses [188]. Similarly, in renal inflammation, soluble biglycan released from the ECM binds to TLR2 and TLR4 on macrophages, promoting the upregulation of CXCL2 and TNF-α, and establishing a feed-forward loop that enhances macrophage infiltration and perpetuates inflammation [189]. Additionally, low-molecular-weight HA fragments accumulate in inflamed tissues, where they interact with TLR2 and TLR4 expression on resident immune cells, stimulating expression of pro-inflammatory cytokines and chemokines [190, 191], and even enhancing interactions between antigen-presenting cells [44]. These extracellular proteins and carbohydrates are considered endogenous danger signals that can activate innate immune cells even in the absence of pathogens.

In cancer, CD8^+^T cells are pivotal for ECM remodeling following paclitaxel (PTX) chemotherapy. Upon activation, these cells exhibit high levels of LOX expression in the spleen and lungs, which is further increased after PTX treatment. LOX facilitates the cross-linking of collagen and elastin, which are essential for ECM remodeling [192, 193]. Natural killer (NK) cell activation can enhance the protein levels of heparanase (HPSE), a heparin-sulfate-degrading enzyme. HPSE activity enables NK cells to degrade the ECM, aid cancer cell invasion, and promote migration across the BM. Conversely, NK cells deficient in HPSE show reduced invasion and migration abilities [194]. HPSEs play a vital role in dendritic cells (DCs). HPSE is produced and sustained in an activated state within the DCs localized on the cellular surface and membranous protrusions. It augments the migratory functions of mature DCs by facilitating ECM degradation. HPSE-mediated ECM degradation not only fosters DC migration but also influences the DC phenotype as they traverse from peripheral tissues to regional lymph nodes, where they present antigenic peptides to T lymphocytes [195].

In summary, in the inflammatory microenvironment and TME, these immune cells actively participate in ECM remodeling, including the secretion of ECM remodeling enzymes, growth factors and cytokines, the promotion of ECM deposition, and the formation of complex interactions with ECM. These interactions form a complex network that plays a key role in tumor initiation, progression, and metastatic dynamics.

## ECM physical properties dictate immune cell fate

The physical properties of the ECM, especially its stiffness, are key determinants in regulating the fate of immune cells. In the TME, the ECM undergoes significant remodeling, and its stiffness, density, and fiber arrangement are changed [196]. These changes not only act as physical barriers to directly affect the migration, activation and function of effector immune cells such as T cells, but also profoundly shape the polarization of macrophages through mechanical signal transduction pathways, usually driving their transformation to the immunosuppressive M2 phenotype [197, 198]. Stiffness, collagen cross-linking, and high-density matrix structure together constitute an immunosuppressive physical microenvironment that limits anti-tumor immune responses and is associated with immunotherapy resistance [199]. This chapter will systematically describe the definition and regulatory mechanism of ECM stiffness, and focus on how ECM stiffness determines the fate and function of immune cells through physical barrier effect and intracellular mechanosensing mechanism.

### What is ECM stiffness?

Stiffness, also known as the modulus of elasticity, is the resistance of a material to deformation in response to a force applied at a slow rate [200]. Abnormal changes in ECM stiffness are associated with many disease states, especially cancer, where the ECM undergoes dynamic remodeling during tumor progression. These changes are reflected in the composition, spatial structure, fiber arrangement direction, and biomechanical properties, which jointly regulate ECM stiffness [196].

The density and arrangement of collagen and elastin are the key determinants of ECM stiffness. Collagen/elastin crosslinking and highly organized matrix fibers are responsible for increased matrix stiffness [201, 202]. LOX is the main enzyme involved in the covalent cross-linking of ECM proteins. Mechanistically, LOX catalyzes the oxidation and deamination of lysine and hydroxylysine residues in collagen and elastin precursors to generate lysine residues. It then reacts with other lysine residues to form cross-links [203]. Lysyl hydroxylase 2 (LH2) specifically hydroxylates lysine residues in collagen terminal peptides. This is crucial for stable cross-linking [204]. LH2 secreted by CAFs induces the cross-linking of hydroxylysine aldehyde-derived collagen in the tumor matrix, thereby increasing tumor matrix stiffness [205]. Elevated ECM stiffness activates cellular responses through mechanical signal transduction pathways, such as the integrin/FAK and YAP/TAZ signaling pathways, thereby driving tumor evolution to a malignant phenotype [206, 207]. Matrix hardness is regulated by oncogenes and tumor suppressor genes. The transcription factors Twist1 and ZEB1 are powerful oncogenes that promote EMT and cancer metastasis. ZEB1 upregulates the expression of LOX and LOXL2 by inhibiting miR-200, thereby promoting collagen crosslinking and matrix hardening [208]. The overexpression of Twist1 not only promotes the transformation of fibroblastic-CAF but also increases matrix stiffness by promoting the expression of type VI collagen α1 chains in CAFs [209]. In addition, tubular smooth muscle cells sense an increase in matrix stiffness through the DDR1/DNMT1 mechanical transduction axis, in which DDR1 is activated in a collagen-independent manner; and DNMT1 is downregulated via ERK-p53 signaling to trigger inflammatory phenotypes, calcification, and arterial stiffness, providing a new mechanism for the intersection of vascular mechanics biology and epigenetics [210].

Within this rigid and hydrated ECM network, various soluble factors, such as growth factors, angiogenic factors, and chemokines, are stored, collectively fostering a persistent inflammatory milieu. This inflammatory environment further promotes the generation of myofibroblasts and macrophages, leading to the excessive deposition of growth factors and ECM proteins. Consequently, ECM stiffness increases, perpetuating a dynamic cycle of ECM remodeling and reinforcement [211, 212].

The emerging techniques for measuring ECM stiffness are shifting from static, ex vivo, single-point assessments toward dynamic, in vivo, and high spatiotemporal resolution approaches. The core idea is to convert ECM stiffness into visual or fluorescent signals, enabling “mechanical visualization”. Traction force microscopy (TFM) is a single-cell force measurement technique that quantifies cellular forces based on cells pulling on their adhesive substrate (the ECM), combined with known material properties of the substrate [213]. Recent advances have extended the application of TFM from two-dimensional (2D) to three-dimensional (3D) microenvironments. 3D TFM allows direct measurement of cellular stresses and pressures within 3D tissues as well as key parameters governing cellular force generation [214].

Another emerging approach, nonlinear stress inference microscopy (NSIM), analyzes tissues in 3D with exceptionally high spatiotemporal resolution [215]. By leveraging the nonlinear stiffening behavior of the ECM, NSIM quantifies the contractile forces exerted by cells on their surrounding matrix, revealing mechanisms that were previously difficult to capture experimentally. In cases where direct in vivo mechanical measurements remain challenging, computational modeling is often employed to uncover the mechanisms underlying morphogenesis [216].

Förster resonance energy-transfer (FRET) sensors consist of donor and acceptor fluorophores. The donor acts as an oscillating dipole that transfers energy to a nearby acceptor with a matching resonance frequency. The FRET signal intensity depends on the distance between the fluorophores, reflecting the extension of the molecular springs and thereby indicating the mechanical tension experienced [217]. For instance, Vuong-Brender et al. used FRET-based sensors to investigate the role of HMP-1/α-catenin in adherens junctions in *Caenorhabditis. elegans*. They observed that the tension on HMP-1 decreased with reduced actomyosin activity, demonstrating mechanosensitivity [218]. Integrating artificial intelligence with multi-physics computational models, combined with high-throughput mechanical and biological data, holds promise for unraveling complex mechanoregulatory networks. Such collaborative approaches will help translate AI-driven discoveries into broadly accessible strategies for combating diseases, such as cancer [219, 220].

### Barrier effects and stromal exclusion of immune cells

The TME encompasses the ECM, which presents a formidable obstacle for immune cell infiltration and hinders their passage. The ECM limits excessive tissue infiltration by immune cells through barrier functions. The alignment, density, and stiffness of collagen, the primary component of the ECM, are crucial factors that influence immune cell migration [14, 199].

#### ECM regulation of T-cell migration and function

During tumorigenesis, augmented cross-linking and increased rigidity of collagen matrices impede the migratory capacity of T cells. Tumor ECM collagen exhibits high heterogeneity, often being tighter around the tumor and looser in the center, with fibers typically oriented perpendicular to the tumor boundary, affecting immune cell migration [221, 222]. For example, CD8^+^ T cells accumulate in the stroma of patients with ovarian and lung cancer. With collagen fibers, the spacing and density limit their contact with tumor cells [197]. Moreover, when T cells pass through narrow spaces, such as those in high-density collagen, their nuclei are compressed and deformed, which can cause the nuclear membrane to rupture. Following rupture, the number of 53BP1 foci increases significantly, indicating the occurrence of DNA double-strand breaks. These breaks lead to reduced cell motility and even cell death [223]. In a 3D collagen matrix culture system, T cells navigate along the collagen network independently of integrin or protease activities [224, 225]. CD8^+^ T cells encapsulated in collagen hydrogels with varying fiber arrangements exhibit enhanced motility when aligned along the fiber axis [224]. The depletion of HA and proteoglycan link protein 1 (HAPLN1), a protein that mediates the connection between HA and proteoglycans, can trigger the alignment of collagen fibers within melanoma cells, thereby hindering the migratory ability of CD8^+^ T cells. HAPLN1 also promotes the infiltration of myeloid-derived suppressor cells (MDSCs) and Tregs [226]. In GC, reduced HAPLN1 expression in CAFs leads to fewer and less dense collagen fibers, promoting ECM remodeling and tumor invasion. Before cancer cell invasion, the ECM is remodeled with radially oriented fibers reorganized in a pattern conducive to invasion. An innovative 3D dual-topographical tumor model surrounding tumor spheroids with radially aligned and circumferentially oriented collagen fibers guides fiber alignment during their assembly using different mechanical forces. Using such a model, research suggests that therapeutic strategies aimed at normalizing the pre-invasive collagen fiber arrangement around tumors could potentially mitigate subsequent invasion [227].

**Fig. 3:**
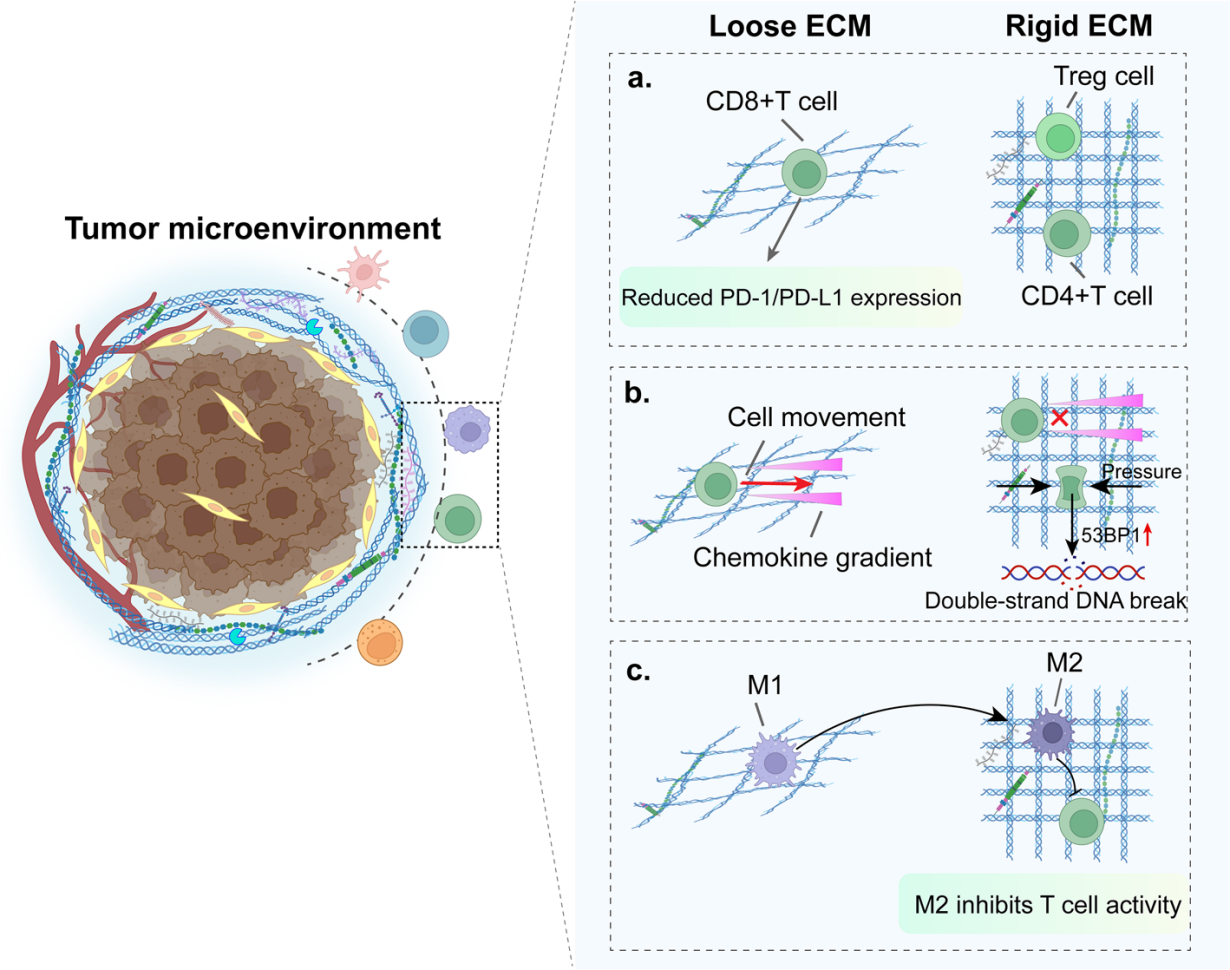
ECM acts as a physical barrier and has implications for the immune cell infiltration phenotype and motility. **a** The impact of ECM density and stiffness on the T cell phenotype; a loose matrix facilitates CD8^+^ T cell support and enhances PD-1 therapy efficacy, whereas a dense, rigid matrix promotes an immunosuppressive phenotype. **b** Influence of matrix properties on T cell motility; in a loose matrix, chemokine gradients promote T cell motility; conversely, in a rigid ECM, T cells lack chemokine-directed motility. **c** The role of high-density ECM in immune regulation drives macrophage polarization from M1 to M2, consequently reducing the attraction and activity of CD8.^+^T cells. ECM: Extracellular matrix; Treg: Regulatory T cell; PD-1: Programmed death 1; PD-L1: Programmed death- ligand 1

Reorganized ECM collagen forms high-density structures that increase ECM stiffness and promote BC metastasis, as revealed by advanced imaging technologies such as small-angle X-ray scattering tensor tomography (SAXS-TT) and X-ray fluorescence computed tomography (XRF-CT) [228]. High-density collagen is correlated with poor prognosis in various cancers [229–233]. In TNBC, high-density collagen promotes Treg infiltration, creating an immunosuppressive TME [230]. A dense ECM also hinders the interactions between DCs and T cells, which are crucial for the immune response to tumors [234]. A study using hydrogel-integrated cultures demonstrated that Jurkat T cells showed lower proliferation on stiffer substrates, which triggered IL-2 secretion [235]. A 3D culture with varying collagen densities indicated that increased matrix stiffness reduces CD8^+^ T cell infiltration in breast tumors, leading to a higher CD4^+^ to CD8^+^T cell ratios and reduced CD8^+^ T cell activity [236] (Fig. 3). Mechanistically, CD4^+^ T cells form complexes with rigid matrix surfaces, thereby suppressing T cell activation [237]. In experiments with 3D collagen matrix cultures, CD8^+^ T cells migrated more swiftly through low-density collagen gels than through high-density gels [238, 239], which was attributed to the smaller pore size of high-density collagen matrices [239].

Nevertheless, elevated ECM stiffness, concomitant with augmented collagen density, was accompanied by a reduction in T-cell migratory velocity. In studies using optically tunable hydrogels, this detection system confirmed that increased ECM stiffness results in diminished T-cell migration irrespective of pore dimensions [240, 241]. In PDAC models, reducing the matrix stiffness significantly enhances the migration and infiltration speed of T cells, leading to an increase in the population of CD8^+^ T cells within the ECM and tumor islets, thereby improving the efficacy of PD-1 therapy [242] (Fig. 3). In lung adenocarcinoma (LUAD) cells, rigid matrices enhance programmed death ligand 1 (PD-L1) expression via actin-dependent mechanisms [243]. Clinically, increased collagen expression and immune checkpoint markers, such as LAIR-1, T-cell immunoglobulin, and mucin domain-containing protein 3 (TIM-3) in patients with melanoma undergoing PD-1 blockade therapy are associated with poor survival, decreased CD8^+^ T cell counts, and increased CD8^+^ T cell subsets [244].

#### ECM stiffness drives immunosuppressive macrophage polarization

As previously mentioned, TAMs contribute to the tumor-promoting ECM through their unique matrix proteinases, guided collagen cross-linking, and deposition of oncotypic ECM components. Conversely, an abnormal ECM regulates the migration, polarization, and function of TAMs.

Macrophages are remarkably diverse and plastic, and their polarization to the M1 or M2 phenotype is strongly influenced by the surrounding microenvironment [245, 246]. Macrophages polarized towards an M1 phenotype manifest a pro-inflammatory state, marked by the expression of cytokines such as IL-12, TNF-α, and inducible nitric oxide synthase (iNOS). M1 macrophages are adept at antigen presentation through the major histocompatibility complex (MHC) molecules. In contrast, M2-polarized macrophages exhibit an anti-inflammatory profile, as evidenced by the expression of markers such as TGF-β, arginase-1 (ARG1), and IL-10 [246, 247]. Interestingly, single-cell sequencing analysis has revealed that subpopulations of TAMs can concomitantly express genes associated with both the M1 and M2 phenotypes, indicating a complex phenotype and transition state between the M1 and M2 phenotypes of TAMs [248]. As the tumor progresses toward malignancy, TAMs predominantly adopt the M2 phenotype, which is characterized by significant immunosuppressive functions within the TME [198]. During monocyte differentiation into macrophages or polarization towards an M2-like phenotype, TAMs interact with the ECM, and particularly with collagen. The mechanical properties and composition of the surrounding collagen significantly influence TAM behavior [249], including migration and immunosuppressive activity [250].

The increase in ECM stiffness within the TME is mainly caused by ECM remodeling. ECM stiffness is a critical biomechanical property that denotes the resistance of the ECM to deformation when subjected to mechanical stress. This significantly affects the activation, polarization, migration, infiltration, cytotoxicity, and antigen presentation of immune cells and thereby has a profound impact on the efficacy of tumor immunotherapy [119, 251]. An increase in ECM stiffness has been linked to the transition of macrophages toward the M2 phenotype. For example, increased ECM stiffness in liver cancer enhances M2 polarization via the integrin β5-FAK/MEK1/2-ERK1/2 pathway in macrophages and activates HIF-1α-induced LOXL2 expression [252]. Culturing BM-derived macrophages (BMDMs) in a low matrix stiffness environment promotes the transition from the M2 to M1 phenotype by modulating the ROS-initiated nuclear factor-kappaB (NF-κB) pathway [253]. Using single-cell RNA sequencing, researchers investigated the influence of matrix stiffness on tumor heterogeneity in both stiff and compliant mouse breast tumors. These findings revealed a notable increase in the proportion of M2-like macrophages within the rigid TME [254]. In addition, BMDMs and TAMs obtained from murine tumors and cultured in high-collagen matrices that mimic tumor tissues exhibited comparable expression of immunosuppressive genes and chemokines. In co-culture experiments, macrophages cultured in high-density collagen exhibited greater efficacy in inhibiting CD8^+^ T cell proliferation and chemotaxis [14] (Fig. 3). In the decellularized stroma of colorectal tumors, a higher density of collagen compared to that present in normal tissue stroma drives monocytes toward M2 polarization, and promotes cancer cell invasion via a mechanism involving CCL18 [255]. βig-h3/transforming growth factor-β-inducible protein (TGFβi) plays an important role in regulating the stiffness of the pancreatic stroma. βig-h3 binds to COL I to form thicker fibers, which promote macrophage conversion to the M2 type and inhibit the proliferation of CD8^+^ T cells, creating a tumor-immunosuppressive microenvironment that accelerates PDAC progression [120].

In summary, ECM acts as a multifaceted regulator of immune responses within the TME. The physical properties of the ECM, including density and stiffness, significantly affect immune cell dynamics, macrophage polarization, and overall tumor immunity. Targeting the ECM and its interactions with immune cells holds promise for developing therapeutic strategies aimed at enhancing anti-tumor immune responses and improving patient outcomes.

## Adhesion molecules mediate the ECM-immune cell interactions

**Fig. 4:**
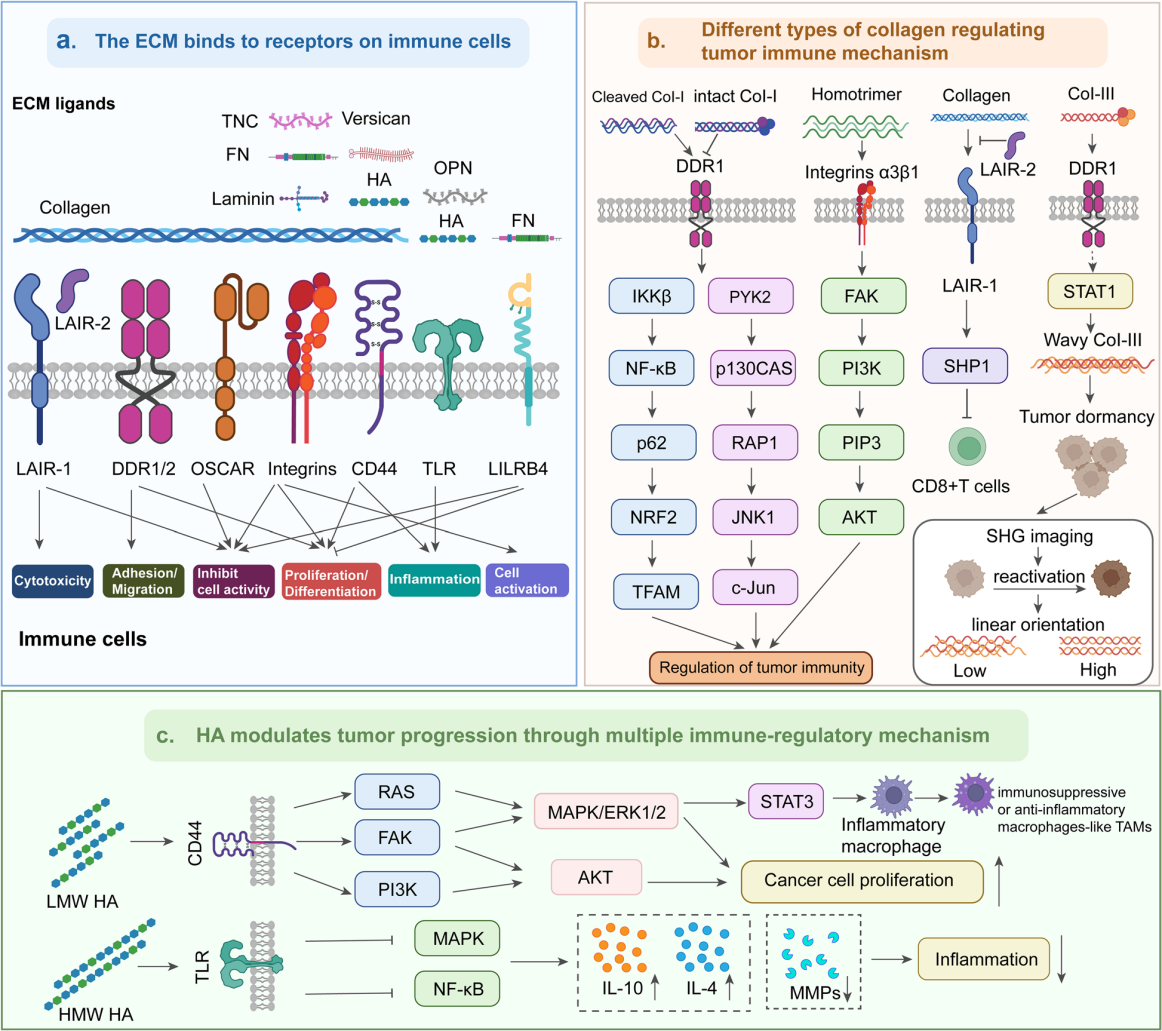
Communication between ECM and immune cells in the TME. **a** ECM, as a ligand, binds to receptors on immune cells to regulate the homeostasis and effector functions of immune cells. **b** Different types of collagen regulate tumor immunity through receptor signal transduction and influence tumor progression. **c** Mechanisms by which different forms of HA influence tumor development via immune regulation. ECM: Extracellular matrix; TNC: Tenascin-C; FN: Fibronectin; HA: Hyaluronan; OPN: Osteopontin; LAIR: Leukocyte-associated Ig-like receptor; DDR: Discoid protein domain receptor; OSCAR: Osteoclast-associated receptor; CD44: Cluster of Differentiation 44; TLR:Toll-like receptor; LILRB4: Leukocyte immunoglobulin-like receptor B4; CoI-I: Collagen Type I; IKKβ: Inhibitor of Nuclear Factor κB Kinase Subunit Beta; NF-κB:Nuclear factor-kappaB; NRF2: Nuclear Factor Erythroid 2-Related Factor 2; TFAM: Mitochondrial Transcription Factor A; PYK2:Proline-Rich Tyrosine Kinase 2; p130CAS: Crk-Associated Substrate p130; RAP1: Ras-Associated Protein 1; JNK1: c-Jun N-Terminal Kinase 1; FAK:Focal adhesion kinase; PI3K: Phosphatidylinositol 3-Kinase; AKT: Protein Kinase B; SHP1: Src homology 2 domain-containing protein tyrosine phosphatase 1:STAT1: Signal transducer and activator of transcription 1; SHG: Second-harmonic generation

The dynamic interplay between cancer cells and adjacent non-malignant host cells, along with vascular and ECM remodeling, is regulated by intercellular contact and paracrine signaling mechanisms. Cell–cell interactions primarily occur through adhesion molecule-mediated binding, membrane protein-receptor engagement, and localized tunneling nanotube-mediated communication. Based on their functional characteristics and regulatory mechanisms, receptors mediating ECM-immune cell interactions can be classified into three major categories: integrins, HA receptors, and receptor tyrosine kinases [256]. Integrins play a central role in immune cell recruitment, migration, and activation at inflammatory or tissue sites through distinctive bidirectional signaling mechanisms [257]. HA receptors such as CD44 recognize HA of varying molecular weights and collaborate with receptors such as TLRs to bidirectionally modulate immune cell migration, activation, and inflammatory responses [258]. Receptor tyrosine kinases, including DDR1/2, are activated upon binding to ECM components and directly phosphorylate downstream signaling molecules, thereby regulating immune cell survival, proliferation, and functional differentiation [259]. As illustrated in Fig. 4, these interactions represent the key mechanisms by which the ECM, acting as a ligand, engages immune cell receptors to mediate cellular communication.

### Integrin adhesion receptors

Integrins are a superfamily of cell adhesion receptors that facilitate physiological processes by binding to ECM ligands and cell surface receptors [260]. Integrins modulate immune cell activity to preserve immune and physiological homeostasis [261, 262]. The complexity of integrin subtypes increases with organismal complexity, culminating in mammals with 18α and 8β subunits. These subunits can assemble into 24 distinct αβ heterodimers, each with unique ECM ligand specificity, including collagen, FN, laminin, and vitronectin [263].

The interaction of collagen with T cells is mediated by α1β1, α2β1, α10β1, and α11β1 integrins. Upon stimulation with the CD3 T cell receptor complex [264], integrins, which are expressed on activated T cells, enhance ECM adhesion [265]. COL I, a potent β1 integrin-dependent co-stimulatory molecule, is more effective than FN in promoting effector T-cell clustering [266]. Specifically, α2β1 integrin activation by COL I has a notable co-stimulatory effect [234]. COL I produced by pancreatic tumor cells is a homotrimer composed of three α1 chains. Due to the lack of the α2 chain encoded by *COLIA2*, this structure participates in regulating the recruitment and function of immune cells by binding to integrins and further activates the integrin α3β1-mediated FAK/AKT/ERK signaling pathway. Ultimately, this promotes the formation of an immunosuppressive microenvironment [267]. Additionally, FN and laminin, through the very late antigen (VLA-4), VLA-5, and VLA-6 integrins, augment CD3-mediated T cell proliferation [268–270].

TNC, a latecomer to immunotherapy, interacts with α5β1 integrins on the surface of T cells and inhibits the cytoskeletal reorganization of actin required for T-cell activation, and thus supports the survival of lymph node metastatic prostate cancer [271]. In glioblastoma, cancer cells secrete exosomes expressing TNC, which targets α5β1 and αvβ6 integrins to inhibit the single chain and activate T cell mechanistic target of rapamycin (mTOR) [272]. Macrophages express various integrins, predominantly the β2 integrin family, but also α2β1 integrins, which are pivotal in macrophage migration and mechanosensing on 3D collagen matrices [273]. α2β1 integrin also facilitates M2 polarization in the Tamm-Horsfall protein-1 (THP-1) monocytic cell line under 3D gel culture conditions [274].

### Discoidin domain receptors

DDRs are receptor tyrosine kinases that specifically bind collagen molecules whose ligands are collagen molecules. Specifically, DDR1 and DDR2 bind to fibrillar collagen; DDR1 preferentially binds to collagens IV and VIII, and DDR2 interacts with collagens I-III, V, and X [275, 276]. DDR1 has six isoforms and is predominantly expressed in cancer and epithelial cells [277, 278]. In contrast, DDR2 is primarily expressed in mesenchymal cells, including fibroblasts and smooth muscle cells [279]. Interestingly, some studies have indicated that T cell receptor (TCR)-activated T cells can induce DDR1 expression. The extracellular domain of DDR1 mechanically binds to collagen and mediates collagen fiber arrangement, potentially impeding T cell infiltration. Consequently, we hypothesized that DDR1 could serve as an intriguing immunotherapy target, potentially enhancing T cell migration into tumors [279].

Research on the effects of DDR1 on macrophage function has been conducted using THP-1 cells expressing enhanced levels of DDR1 [280, 281]. Notably, high expression of DDR1 a in THP-1 cells mediates infiltration and migration within 3D collagen matrices [280]. Additionally, in monocytes cultured on collagen-coated surfaces, overexpression of DDR1b results in increased levels of inflammatory markers such as IL-1β, IL-8, monocyte chemoattractant protein-1 (MCP-1), and macrophage inflammatory protein-1 alpha (MIP-1α). Levels of these markers were found to be significantly elevated in DDR1b-overexpressing monocytes cultured on collagen-coated surfaces. These markers trigger the MAPK/NF-κB pathway, culminating in macrophage activation during inflammatory processes [281]. Interestingly, cleaved COL I (cCOL I) and intact COL I (iCOL I) exert opposing effects in PDAC. Specifically, cCOL I promotes tumor growth by activating the DDR1/NF-κB/p62-NRF2 signaling axis, whereas iCOL I induces DDR1 degradation and suppresses PDAC progression. This highlights how the functional role of COL I in PDAC is determined by its structural form [282]. Furthermore, cCOL I modulates tumor immunity via the DDR1/PYK2/JNK1/c-Jun pathway, whereas iCOL I counteracts this effect [256]. ECM proteomics studies have revealed that dormant cancer cells assemble an ECM niche enriched in type III collagen. By binding to type III collagen, DDR1 activates STAT1 signaling, which promotes dormancy and upregulates COL3A1 expression in disseminated tumor cells, thereby reinforcing the pro-dormancy ECM niche. To further characterize the ECM architecture surrounding dormant versus reactivated tumor cells, Di Martino et al. employed multiphoton second-harmonic generation (SHG) imaging to assess collagen fiber organization. They found that dormant tumor cells were embedded in an ECM dominated by wavy collagen fibers with low linear alignment, whereas the reactivation of cancer cells was associated with highly aligned linear collagen fibers [283].

In a 3D collagen culture medium, DDR2 expression by human primary neutrophils induces increased MMP secretion. This results in the formation of local gradients of collagen-derived chemotactic peptides that regulate neutrophil directionality [284].

Notably, in PDAC and BC, collagen-activated DDR1 upregulates CXCL5. This upregulation leads to the infiltration of TANs and the formation of NETs [285] and plays a role in promoting lung metastasis of BC cells and influencing Treg differentiation and immune infiltration in BC [286].

Furthermore, DDRs contribute to immune checkpoint blockade (ICB). DDR gene signatures define distinct T cell tumor infiltration patterns and correlate with the survival of urinary tract cancer patients with UTC treated with PD-L1 checkpoint inhibitors. Remarkably, these signatures can also stratify immunotherapy outcomes in other cancer types, such as melanoma [287]. Targeting DDR2 significantly enhances the response to ICB with PD-1 inhibitors, as demonstrated in an in vivo pan-cancer model [288].

Interactions between the ECM and the immune system are remarkably evolutionarily conserved among vertebrates. This conservation was particularly evident in the binding mechanism between DDR1 and collagen. The DDR1-collagen interaction mode is highly conserved across vertebrates such as humans and mice. Key structural features, including the DS domain and the ATP-binding, have remained largely unchanged throughout evolution, underscoring strong structural and functional conservation [289].

### LAIR and OSCAR

Coinhibitory leukocyte-associated LAIR-1 is an immunosuppressive transmembrane receptor that features two immunoreceptor tyrosine-based inhibition motifs (ITIMs) within its intracellular domain. It negatively regulates immune cell function by recruiting Src homology 2 domain-containing phosphatase-1(SHP-1) and SHP-2 [290]. When expressed in myeloid cells, T cells, NK cells, and other immune cell subsets [291], LAIR-1 increases disease progression in LUAD, low-grade glioma (LGG), chronic lymphocytic leukemia, BC, and OSCC. High LAIR-1 levels are often associated with poor prognoses [291–295]. Collagen, particularly COL I, is a high-affinity ligand for LAIR-1 that can bind to immune cell surfaces and trigger inhibitory signals [296, 297]. In cancer, the interaction between collagen and LAIR-1 induces CD8^+^ T-cell exhaustion and suppresses lymphocyte activity via Src homology 2 domain-containing protein tyrosine phosphatase 1 (SHP-1) signaling [244]. Conversely, overexpression of LAIR-2 or its inhibition can negate the inhibitory effects of LAIR-1. Indeed, LAIR-1 immunosuppression, induced by its interaction with collagen, can be reversed by the decoy receptor LAIR-2 (CD306, a soluble inhibitor of LAIR-1), making drug-resistant lung tumors more susceptible to PD-1 blockade. The binding affinity of LAIR-2 surpasses that of LAIR-1 [244, 298, 299]. The COL I fragments produced by MMP1 and MMP9 compromise T-cell function through LAIR-1, leading to T-cell suppression. However, the LAIR-2-Fc recombinant protein can block LAIR-1 binding to collagen, thus enhancing cytotoxic T-cell infiltration and function and facilitating cancer cell elimination during the anti-tumor immune response [300, 301]. Additionally, the LAIR-2-Fc fusion protein amplifies the anti-tumor effects of PD-1/PD-L1 checkpoint blockade therapy, thereby rejuvenating T-cell vitality [301]. Therefore, the interplay between LAIR-1, LAIR-2, and their respective ligands merits further exploration to develop therapeutic agents that can circumvent tumor evasion by the immune system.

LAIR-1 is also present in most myeloid cells in cancer, including monocytes and macrophages, and plays a role in immunosuppression [302]. In monocytes, LAIR-1 interaction with agonistic antibodies impedes TLR-induced signal activation [303]. Recent studies have suggested that activating LAIR-1 with the agonist monoclonal antibody (mAb) NC525 induces leukemic blast cell death in acute myeloid leukemia (AML). These findings highlight the potential of LAIR-1-targeted therapies as a promising approach for the treatment of advanced myeloid malignancies, offering an immunotherapeutic strategy for enhancing antileukemic responses [304]. The first component of this system, C1q, contains a collagen-like domain. C1q and its collagenous tails engage LAIR-1 and LAIR-2, inducing the phosphorylation of ITIMs in monocytes. LAIR-2 can counteract C1q-mediated inhibition of monocyte-DC differentiation and plasmacytoid DC production of IFN-α [305].

LAIR-1 is primarily expressed at high levels in non-classical monocytes, but is also expressed in classical monocytes and TRMs. COLEC12, which possesses a collagen-like domain, was recently identified as a binding partner of LAIR-1, and is involved in modulating the viability, multiplication, and specialization of monocytes and pulmonary interstitial macrophages. This study also highlighted the link between BM-specific tumor cell apoptosis and melanoma lung metastasis [306]. A deeper understanding of the ECM and LAIR-1 governing the behavior of myeloid and T cells is crucial for targeted therapy of solid tumors and hematological malignancies.

Osteoclast-associated receptor (OSCAR) is an immune receptor that binds to collagen and shares a gene family with LAIR-1. Associated with Fcg receptor (FcR)-γ, OSCAR features an immunoreceptor tyrosine-based activation motif (ITAM) in its cytoplasmic tail, a signaling cascade culminating in cell activation [307]. OSCAR have been detected not only in osteoclasts but also on macrophages, monocytes, and monocyte-derived DCs. Although the role of OSCAR in cancer remains largely unexplored, it is known that OSCAR mRNA levels are elevated in most cancer types, and their expression is reduced by NK cell infiltration in the suppressive immune microenvironment, highlighting its potential oncogenic role and suggesting that targeting OSCAR could help reverse this state in future therapies [308].

### CD44, TLR, and LILRB4

CD44, a transmembrane receptor widely expressed in immune and tumor cells, interacts with key ECM components like HA, FN, and laminin. The HA-CD44 interaction serves to modulate immune responses and promote tumor progression. Excessive HA accumulation within the TME contributes to immune suppression by forming a dense ECM network that limits drug penetration and effector immune cell infiltration [309, 310]. HA also regulates immune cell function by activating DCs and inducing T-cell proliferation rather than directly engaging CD44 receptors on T cells [311]. In B cells, HA-CD44 interactions are upregulated in melanoma 2 (AIM2) and signal transducer and activator of transcription 3 (STAT3), promoting B-cell activation and proliferation via the HA/CD44/AIM2 pathway, suggesting a potential immunotherapeutic target [312, 313]. In a murine BC model, the HA/CD44/ERK1/2/STAT3 pathway critically drives in the HA-stimulated formation of immunosuppressive or anti-inflammatory macrophage-like TAMs [314]. Additionally, HA-CD44 signaling influences macrophage polarization, shifting them toward the M2 phenotype, which supports tumor progression [26]. Within the TME, ECM remodeling is closely linked to abnormal structure and function of HA, whose effects vary depending on its molecular weight [315]. HA can promote anchorage-independent growth of cancer cells by activating the PI3K/Akt survival pathway. In particular, lower molecular weight HA (LMW-HA) further stimulates tumorigenesis and progression by binding to CD44 and activating the RAS/PI3K/Akt signaling cascade. This process also induces EMT and facilitates metastatic dissemination. Notably, such pro-tumorigenic mechanisms can be inhibited by HA oligomer blockers [316].

In addition to CD44, HA engages TLRs, particularly TLR2 and TLR4, to trigger the production of inflammatory cytokines by macrophages [317]. High molecular weight HA (HMW-HA) can suppress MAPK and NF-κB signaling pathways by binding to TLR, exert anti-inflammatory effects through the induction of IL-4 and IL-10, and reduce MMP activation [316]. Versican, another ECM component that binds CD44, also interacts with P-selectin glycoprotein ligand-1 (PSGL-1) and TLRs on immune cells, leading to the activation of NF-κB, IL-6, and TNF-α, thereby amplifying inflammation and modulating immune responses in tumors [318, 319]. OPN further contributes to immune regulation through the TLR pathway, where intracellular OPN interacts with MyD88 to modulate TLR signaling, suppress inflammatory responses, and prevent excessive immune activation in liver cancer [320–322].

Leukocyte immunoglobulin-like receptor B4 (LILRB4), also known as IL-1 receptor-like protein 3 (ILT3), is a member of the LILR family of inhibitory receptors found in myeloid cells that transmits inhibitory signals through ITIMs [323]. FN, a key ECM component, functions as a ligand for LILRB4 and promotes immunosuppressive phenotypes in myeloid cells. Upon FN-LILRB4 engagement, myeloid cells exhibit reduced chemokine production, leading to decreased T-cell recruitment and impaired T-cell proliferation. Additionally, FN exposure dampens DC activation by lowering their responsiveness to FcR signaling, further contributing to immune suppression in the TME [324].

More broadly, ECM components such as collagen, FN, laminin, and HA regulate immune responses in solid tumors by interacting with immune cell receptors, including integrins and DDRs. These ECM-immune interactions are pivotal in shaping the immunosuppressive tumor landscape, highlighting the need to investigate ECM-mediated immune modulation as a potential therapeutic strategy.

## Therapeutic strategies for tumor ECM-mediated immunosuppression

Given the crucial role of the ECM in constructing the immunosuppressive TME, targeting the ECM has become a key strategy to overcome immune therapy resistance. Current intervention methods mainly focus on four aspects: directly targeting the key components of the ECM (such as collagen, hyaluronic acid) and their cross-linking enzymes (such as LOX) to degrade the physical barrier or inhibit their abnormal synthesis [325, 326]; using nanotechnology to deliver drugs or enzymes to improve drug penetration and reshape the TME [327–329]; blocking the interaction between the ECM and inhibitory receptors on the surface of immune cells (such as LAIR-1, LILRB4) to relieve their inhibition of immune cells [330, 331]; modifying adoptive cell therapies (such as CAR-T, CAR-M) so that they can actively secrete matrix-degrading enzymes or target matrix cells, thereby breaking through the ECM barrier [332, 333]. Despite challenges such as specificity, timing, and safety, combining ECM-targeting strategies with existing immunotherapies shows a broad prospect for enhancing anti-tumor immune responses.

### Targeting the primary components of the ECM

In a wide range of solid tumors, in which up to 80% of patients fail to respond to PD-1/PD-L1 blockade, the TME consistently exhibits upregulation of ECM-related genes and abnormal accumulation of ECM components. Consequently, the ECM barrier is no longer an isolated biological phenomenon but rather a common and central mechanism underlying clinical resistance across multiple cancer types [153].

As a key regulator of the TME, the ECM directly interacts with immune cells, influencing their function and shaping immune responses. Given its profound role in tumor progression and immune modulation, the ECM has emerged as a therapeutic target for solid tumors, and numerous strategies have been explored to modify its structure and function [334].

**Fig. 5:**
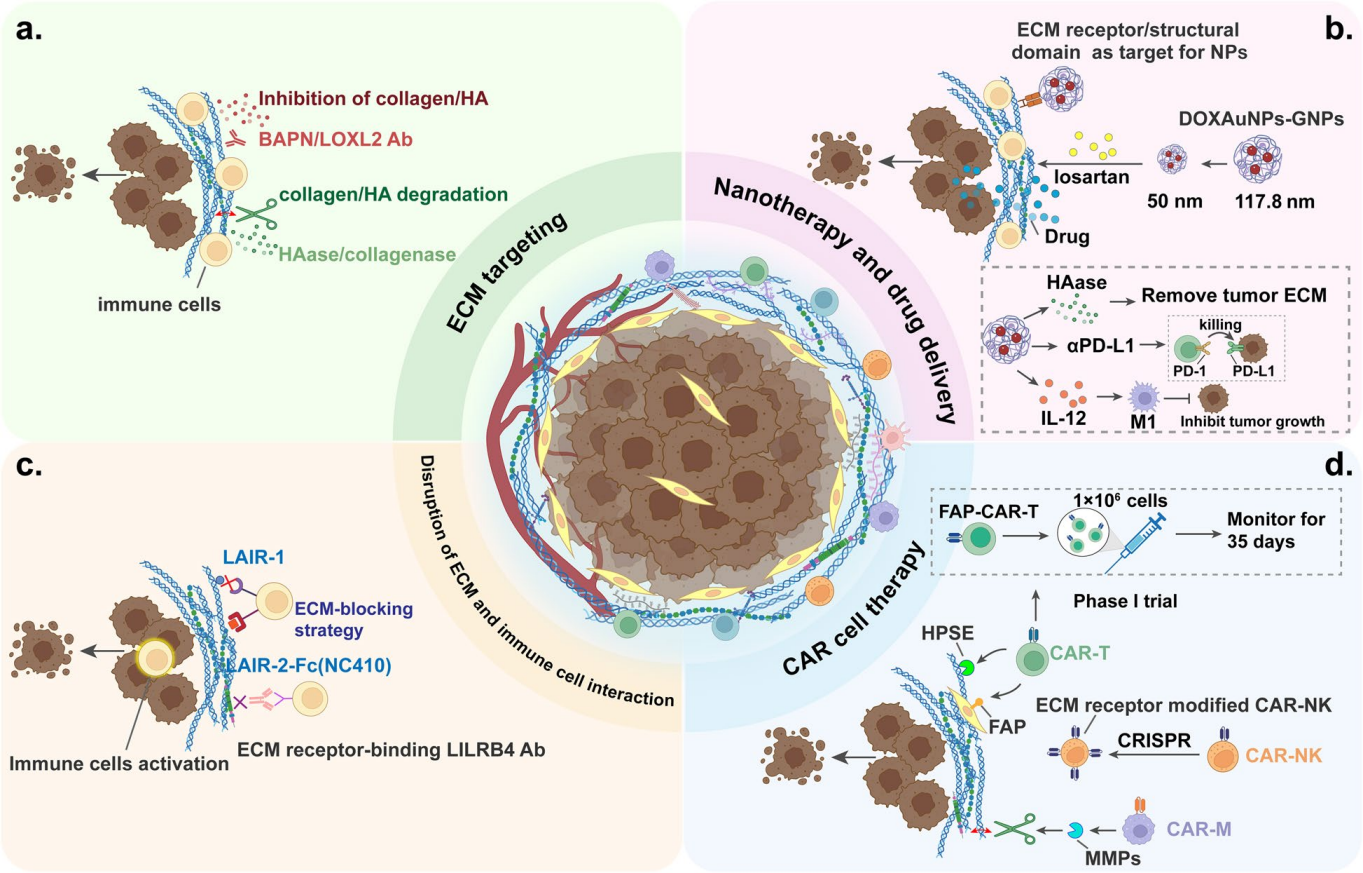
Therapeutic Strategies to Counteract ECM-mediated immunosuppression in TME. **a** Utilizing agentssuch as collagenase and hyaluronidase for ECM degradation, and LOX inhibitors to suppress ECM synthesis. **b** Employing ECM component-targeted nanoparticles topenetrate physical barriers. **c** Blocking interactions between ECM and immune cells. **d** Implementing CAR cell engineering for the treatment of solid tumors. HA: Hyaluronan; BAPN: β-Aminopropionitrile; LOXL: Lysyl oxidase-like proteins; ECM: Extracellular matrix; NPs: Nanoparticles; PD-1: Programmed death 1; PD-L1: Programmed death-ligand 1; IL-12: Interleukin-12; LAIR: Leukocyte-associated Ig-like receptor; FAP: Fibroblast activation protein; CAR: Chimeric antigen receptor; HPSE: Heparanase; MMP: Matrix metalloproteinase; CRISPR: Clustered Regularly Interspaced Short Palindromic Repeats

For example, the use of collagenases or hyaluronidases may enhance the distribution of therapeutic drugs [325]. BAPN, a potent LOX inhibitor, reduces highly cross-linked and stiffened collagen in BC and has proven effective in decreasing ECM stiffness, serving as a prime example of TME remodeling to enhance drug penetration [326]. In osteosarcoma, BAPN inhibits the Wnt/c-Fos/AP-1 pathway, creating a positive feedback loop with LOXL2-induced collagen cross-linking [335]. However, BAPN administration may be toxic; thus, local treatment is required. BAPN inhibition improves the results of experimental liver fibrosis, demonstrating the significant role of LOX family members in stabilizing fibrotic collagen fibers and promoting tissue hardness [131]. In one study, an inhibitory monoclonal antibody (AB 0023) was used to target LOXL2 and effectively prevented the development of primary cancer, with demonstrated efficacy and safety in both primary and metastatic xenograft models as well as in liver and pulmonary fibrosis models, surpassing BAPN [336]. Simtuzumab, an antibody directed against LOXL2, diminishes collagen cross-linking and has been used to treat colorectal carcinoma and hepatic fibrosis [337, 338]. LOXL2 inhibitors, combined with OSM-mediated signaling inhibitors secreted by TAMs, synergistically reduce the metastatic potential of PDAC cells [178]. HA can also be enzymatically degraded. A Phase II clinical trial of PEGylated human hyaluronidase (PEGPH20) combined with standard gemcitabine and nab-PTX chemotherapy demonstrated clinical benefits in patients with pancreatic cancer [339]. However, PEGPH20 combined with the PD-L1 inhibitor atezolizumab showed no clinical activity as a second-line treatment in patients with advanced PDAC or gastric adenocarcinoma who progressed during or after chemotherapy [340]. Other alternative treatments include targeting HA anabolic inhibition by using glutamine-fructose aminotransferase 1 (GFAT1), a pivotal rate-limiting enzyme in the HA synthesis pathway. The use of a glutamine-derived small-molecule analog targeting GFAT1 has been shown to diminish the self-renewal capacity and metastatic potential of cancer cells, making pancreatic cancer more sensitive to anti-PD-1 treatment and prolonging survival [341] (Fig. 5).

To date, strategies to enhance ECM degradation or inhibit ECM synthesis have not yielded significant clinical results and broad-based approaches are likely to encounter persistent challenges. Interventions aimed at blocking ECM synthesis must commence early in tumor development because their efficacy diminishes in late-stage cancers characterized by a preformed dense ECM, which offers no therapeutic benefits. Additionally, the complexity of the ECM, which comprises multiple components and is governed by various factors, leads to nonspecific targeting, which can result in off-target effects, side effects, and adverse reactions. Moreover, although matrix degradation can decrease ECM stiffness and enhance immune infiltration, it may inadvertently release excessive cytokines and growth factors, thereby accelerating tumor progression. In the future, the development of a combination therapy strategy targeting the ECM may be the most reasonable option.

### Nanotherapy and drug delivery

Nanoparticles (NPs) enhance the anti-tumor immune response by reshaping the physical barrier and biochemical microenvironment of the ECM, overcoming drug delivery obstacles, and collaborating with immunomodulators to reprogram the immunosuppressive TME [327–329]. NPs that specifically target ECM components are the optimal choice for enhancing tumor tissue penetration and drug delivery. For instance, NPs modified with collagenase IV and loaded with doxorubicin exhibited superior drug penetration compared to their unmodified counterparts [342]. Additionally, the overexpression of the HA-specific receptor CD168 in metastatic tumors is an ideal target for HA nanogels [343]. The extra-domain B (EDB) of FN and the fibrinogen-like globe (FBG) domain of TNC, targeted by the targeting peptide (PL1), significantly inhibited tumor growth and improved survival in mice, demonstrating that ECM protein domains are viable therapeutic targets for drug delivery [344] (Fig. 5).

Moreover, IL-2 immune cytokines in nanobody-delivered systems delay tumor growth [345]. The BM, which is a crucial ECM structure, can confine NPs within the subendothelial space, thereby creating an NP reservoir. Recent studies have revealed that neutrophil infiltration guided by activated platelets disrupts the BM barrier of tumor blood vessels, releasing trapped NPs in a burst, thereby facilitating nanomedicine penetration through this immune-coordinated mechanism [346].

In HCC, NPs encapsulating HAase, IL-12, and αPD-L1 antibodies are incorporated into a polymer/calcium phosphate (CaP) hybrid system that is sensitive to both pH and MMP-2. The acidity of tumors triggers the dissolution of CaP, which in turn enables the targeted release of IL-12 and ECM-degrading HAase, thereby enhancing tumor infiltration by cytotoxic T lymphocytes (CTLs) and promoting their proliferation. The in situ release of αPD-L1, induced by elevated levels of MMP-2, counters the ability of tumor cells to evade destruction by CTLs, and IL-12-incorporated nanomedicine induces M1 polarization in TAM. This sensitive nanomedicine design offers a promising strategy for delivering protein-based immunotherapeutics, and can potentially be expanded to treat other ECM-rich tumors [347]. EVs deliver molecular components (including nucleic acids and proteins) that can alter the physiological state and biological functions of recipient cells, thereby promoting tumor cell growth, metastasis, angiogenesis, and drug resistance [348, 349]. Cellular nanovesicles (NVs) are effective and abundant alternatives to artificially manufactured EVs and simulate natural EVs. NVs retain the high biological penetration of EVs, target the delivery of therapeutic molecules to break through the ECM barrier, and reprogram the immunosuppressive microenvironment, thereby providing a new strategy for overcoming tumor stromal drug resistance. For instance, NVs enhance the anti-tumor immune response by targeting circulating tumor cells (CTCs) and resected tumor cells, and reprogramming M2-type TAMs into pro-inflammatory M1 phenotypes [350]. Nanoparticulate drugs must traverse the dense ECM to penetrate deep into tumor tissues. Thus, reducing the ECM by inhibiting its formation or accelerating its degradation can lower the transport resistance and enhance NP penetration. He et al. developed TME-responsive NPs (DOX-AuNP-GNPs) that shrink from 117. 8 nm to 50 nm under acidic tumor conditions to improve tissue penetration. However, even after size reduction, these NPs failed to efficiently penetrate the dense tumor ECM. Following losartan pretreatment, which significantly reduces intratumoral collagen, the DOX-AuNP-GNPs formulation exhibited markedly improved tumor penetration and therapeutic efficacy [351].

In the TME, conjugates, such as collagenase-bromelain, have been shown to enhance drug penetration. Bromelain can be covalently linked to an HA peptide, enabling specific targeting of the tumor ECM. Intravenous administration of collagen type IV-binding peptide (C4BP) conjugated to HA (HA-C4BP) results in higher tumor accumulation in 4T1 tumor-bearing mice compared to unmodified HA. Thus, the systemic delivery of the C4BP-HA-bromelain conjugate (C4BP-HA-Bro) enhances the anti-tumor efficacy of DOX-loaded liposomes, likely through the reduction of collagen fiber density and length and improved intratumoral distribution of doxorubicin [352].

Decellularized extracellular matrix (dECM) has emerged as a promising strategy to overcome the challenges associated with NP-based approaches in organoid culture. Tissue-specific dECM scaffolds are increasingly used to establish organoid-based disease models [353]. For instance, Chen et al. developed a liver-derived dECM (L-dECM) scaffold to construct HepG2-based tumor organoid-like tissue models. The L-dECM scaffold not only enhanced liver function, but also promoted EMT, an initial step in tumorigenesis triggered by microenvironmental signals. Moreover, this model displays distinct drug sensitivity profiles, supporting its utility for in vitro anticancer drug screening and EMT-focused research [354].

Nanocarriers face multiple limitations in penetrating tumors with a highly heterogeneous ECM. NP diffusion is not only restricted by the ECM pore size but is also influenced by the surface charge. Charged ECM components, such as positively charged collagen and negatively charged HA, can electrostatically adsorb NPs, leading to their entrapment within the matrix and further impeding deep tumor penetration [355]. Additionally, ECM heterogeneity often coincides with abnormal tumor vasculature and impaired lymphatic drainage, resulting in elevated interstitial fluid pressure. This high-pressure environment hinders both the extravasation of NPs from the blood vessels and their convective transport within the tumor, creating a "physical barrier" [356]. Metabolic abnormalities in TME, including hypoxia and acidosis, are closely linked to an immunosuppressive state. These factors not only reduce NP penetration efficiency, but may also induce drug resistance in tumor cells, further compromising treatment outcomes [357]. Therefore, future strategies should focus on ECM remodeling, optimization of the NP size and surface charge, and combinations with microenvironment-modulating approaches to overcome these limitations.

### Targeted therapy for the interaction between ECM and immune cells

Targeted therapies that disrupt interactions between the ECM and immune cell receptors offer a promising avenue for cancer treatment. For example, recombinant LAIR-2-Fc fusion proteins, such as NC410, have been shown to promote tumor growth in humanized mouse models [300]. NC410 acts as a LAIR-1 antagonist that binds to collagen with high affinity and blocks LAIR-1 signaling [358, 359]. However, recent studies have indicated that blocking LAIR-1 with NC410 alone does not sufficiently control tumor-immunocompetent mice and that a combined approach targeting both PD-L1 and LAIR-1 is more effective in reducing tumor growth [360].

Upon stimulation of T cells via NC410, there is a detectable increase in specific collagen degradation products in vivo, including fragments of type IV collagen that are cleaved by granzyme B and type VI collagen that is broken down by MMPs [361]. LAIR-2 functions as a decoy protein that disrupts LAIR-1-collagen interaction, highlighting its potential as a therapeutic target to counteract ECM-mediated immune suppression. Blocking this interaction may enhance T-cell infiltration and activation, making it a promising strategy for tumor immunotherapy. Similarly, LILRB4 has emerged as an immunotherapeutic target for solid tumors. Monoclonal antibodies (MoAbs) or inhibitors targeting LILRB4 have shown the potential for reprogramming tumor-associated myeloid cells, thereby enhancing T-cell recruitment and activation within the TME. This approach may improve the response to ICB therapy, offering a new avenue for overcoming tumor immune resistance [330, 331, 362]. PRTH-101 has demonstrated the ability to inhibit DDR1 phosphorylation, disrupt collagen-mediated cell adhesion, prevent DDR1 shedding from the cell surface, and effectively break down the ECM barrier in BC. The disruption of CD8^+^ T cell infiltration prevents immune evasion [363]. Additionally, combining a DDR2 inhibitor, such as dasatinib, with PD-1 blockade has been shown to reduce tumor burden [288]. It is important to note that ECM receptors, such as LAIR, LILRB4, and DDR, are not exclusively expressed on immune cells but also in various non-immune cell types. Therefore, targeted blockade must be carefully designed to avoid off-target effects that can disrupt homeostatic functions, such as the interaction between collagen and LAIR-1 in lung interstitial macrophages [306]. The common lymphatic and vascular endothelial receptor-1 (Clever-1), a multifunctional scavenger receptor, facilitates tumor immune escape by enhancing the immunosuppressive activity of TAMs. Genetic or pharmacological disruption of Clever-1 remodels the metabolic profile of TAMs, leading to enhanced glycolytic flux and elevated mTOR signaling. This shift drives TAM repolarization toward a pro-inflammatory M1-like phenotype, which in turn promotes CD8^+^ T-cell-mediated anti-tumor immunity [364]. Moreover, Clever-1 inhibition downregulates PD-L1 expression in both TAMs and tumor cells, thereby attenuating the key “immune brake” mechanism exploited by tumors. Consequently, the combination of Clever-1 blockade with anti-PD-1 therapy demonstrated additive or synergistic effects in breast and colorectal cancer models characterized by high PD-L1 expression and active immune infiltration. In contrast, this combination strategy fails to confer significant benefits in immunologically cold lung cancer models [365].

Furthermore, by integrating multi-omics data with machine learning algorithms, Geng et al. developed an AI-driven prognostic and immunotherapy response model for ovarian cancer based on 15 ECM-related genes. This model accurately predicts patient outcomes and responses to immunotherapy. The study revealed that patients in the low-risk group exhibited greater sensitivity to immunotherapy associated with the RYR2 gene, indicating that they might benefit more from PD-1/PD-L1 inhibitors. These findings provide new biomarkers for the personalized treatment of ovarian cancer [366].

Endostatin, a proteolytic fragment of collagen XVIII, frequently induces drug resistance during antiangiogenic therapies. Studies have shown that in xenograft mouse models, endostatin triggers intratumoral hypoxia, leading to an expansion of ALDH⁺ lung cancer stem-like cells (CSLCs) [367]. Additionally, endostatin promotes the recruitment of TAMs, MDSCs, and Tregs, which secrete various cytokines and growth factors that enhance CSLC proliferation. Therefore, combining endostatin with agents targeting CSLCs may improve clinical efficacy [368].

### Treatment of solid tumors with CAR T-cell therapy

Over the past decade, chimeric antigen receptor (CAR) T-cell therapy has demonstrated considerable efficacy in the treatment of hematological malignancies, yet has shown limited efficacy against solid tumors. The limited infiltration of CAR-T cells and the immunosuppressive nature of the TME present major obstacles to their effectiveness in treating solid tumors [369]. CAR-T cells have been engineered to secrete matrix-degrading enzymes that enhance their effectiveness, disrupt the physical barriers of solid tumors, and improve their migration and infiltration abilities. For instance, CAR-T cells engineered to secrete HPSE can degrade tumor ECM and facilitate tissue penetration [332]. Studies have shown that fibroblast activation protein (FAP), a specific marker of CAR-T cells targeting CAFs, can ablate CAFs, reduce connective tissue hyperplasia, and promote T cell infiltration and anti-tumor activity [370, 371] (Fig. 5). The development of OncoFAP, a high-affinity ligand for FAP, has opened new avenues for CAR-T cell applications in the clinical setting [372].

A Phase I clinical trial (NCT01722149) was conducted for malignant pleural mesothelioma (MPM), a rare and highly aggressive malignancy originating from the pleural tissue. In preclinical studies, FAP-targeted CAR-T cells have demonstrated antigen-specific activity both in vitro and in vivo, suppressing the growth of intraperitoneally implanted FAP-positive human MPM cells in mice and significantly prolonging survival [373]. Twenty-one days before clinical treatment, CD8^+^ T cells were isolated from the peripheral blood of the patient and were transduced with a second-generation FAP-targeted CAR via a retroviral vector. A single dose of 1 × 10^6^ CAR-T cells was then administered intrapleurally via the pleural effusion. After over 35 days of continuous monitoring, the systemic expansion of FAP-CAR-T cells was detected in the blood of all three patients. Two patients developed postoperative thromboembolic events, which were managed and resolved; these were assessed as likely related to cancer progression, chemotherapy, or catheter manipulation, rather than the CAR-T therapy itself. The trial demonstrated a favorable initial safety profile and efficacy trend, supporting the further clinical development of FAP-CAR-T therapy. Recent studies have increasingly explored combinations of strategies. One ongoing trial aims to evaluate the synergistic effects of combining FAP-targeted CAR-T cells with Nectin4-targeted CAR-T cells (NCT03932565), expanding its potential applicability across multiple solid tumors [374]. Meanwhile, Claudin18. 2 (CLDN18. 2) has emerged as a novel therapeutic target owing to its frequent overexpression in various malignancies, particularly gastrointestinal cancers [375]. Studies have indicated that sequential administration of FAP- and CLDN18. 2-targeted CAR-T cells enhances the efficacy of PDAC. Specifically, the initial infusion of FAP-CAR-T cells to deplete CAFs in the TME, followed by CLDN18. 2 CAR-T cells to directly eliminate tumor cells, resulted in superior anti-tumor activity compared with the concurrent administration of both CAR-T products [376].

CAR-NK cells offer several advantages over CAR-T cells, including lower toxicity, the synergistic effects of multiple activating receptors, and the potential for creating universal “off-the-shelf” therapies. These cells are currently being explored in preclinical studies and clinical trials [377, 378]. CRISPR gene editing to knock out inhibitory ECM receptors, such as LAIR-1 or LILRB4, is a promising method for enhancing the efficacy of CAR-NK against ECM-rich solid tumors while minimizing off-target effects [379] (Fig. 5). Recently, CAR-macrophages (CAR-Ms) have emerged as potential drivers of anti-tumor immunity in solid tumors by exploiting the ability of macrophages to produce MMPs for ECM degradation [369]. CAR-147, a type of CAR macrophage, targets HER2-positive tumor cells and activates MMP expression, thereby disrupting the tumor ECM and promoting T-cell infiltration [333] (Fig. 5). Despite the challenges faced by CAR-T, CAR-NK, and CAR-M therapies in the treatment of solid tumors, their potential for ECM remodeling remains an underexplored area with significant research prospects.

## Clinical trials targeting ECM in inflammation and cancer

Numerous compounds targeting ECM components and remodeling processes have been screened in preclinical studies, and their efficacy against various tumors and related inflammation has been confirmed both in vitro and in vivo [380]. However, key issues such as safe dosage, toxic side effects, and clinical benefits of these compounds in humans require further clarification through clinical trials. Therefore, below we discuss targeted drugs that have already been tested in patients. ECM-targeting strategies include pre-targeted degradation of ECM components (e. g., HA and collagen), inhibition of collagen cross-linking, and reduction of ECM stiffness and density to enhance therapeutic efficacy, as detailed in Table 2.

**Table 2:** Clinical trials of targered ECM therapy

Mechanism	Therapeutics	Cancer type(s)	Phase	Trial no	Status	Outcome	Target	Combination standard of care therapy
Targeting ECM Components	Losartan	Pancreatic Cancer	II	NCT01821729	Unknown status	Significantly improves the rate of negative margin (R0) resection in patients with PDAC	Angiotensin II receptor	FOLFIRINOX
			I	NCT04106856	Recruiting			HRT
	GC1008	Renal Cell Carcinoma or Malignant Melanoma	I	NCT00356460	Completed		TGFβ	No combination
	LY2157299	Rectal Cancer	II	NCT02688712	Recruiting	Improved the complete response rate to 32% and was well tolerated		chemotherapy + radiation
	Simtuzumab	Metastatic Pancreatic Adenocarcinoma	II	NCT01472198	Complete	The addition of simtuzumab to gemcitabine did not improve clinical outcomes	LOXL2	Gemcitabine
	PAT-1251	Healthy Adult	I	NCT02852551	Complete	This compound was well tolerated		No combination
	PEGPH20	Pancreatic Cancer	II	NCT01839487	Completed	This compound boosts PFS in untreated metastatic pancreatic cancer patients	HA	Nab-Paclitaxel + Gemcitabine
		Morpheus-Pancreatic Cancer	Ib/II	NCT03193190	Completed	Atezolizumab in combination with PEGPH20 is tolerable		Atezolizumab + PEGPH20 Atezolizumab + PEGPH20
		Gastric, Gastroesophageal Junction Cancerr, Esophageal Cancer	Ib/II	NCT03281369	Active			
		Pancreatic Cancer	I/II	NCT01959139	Completed	This compound underperformed for all efficacy endpoints (OS/PFS/RR) and had a significant negative impact on survival		chemotherapy
		Pancreatic Ductal Adenocarcinoma	III	NCT02715804	Terminated			nab-paclitaxel + gemcitabine
	RO5429083	Metastatic and/or Locally Advanced, CD44-Expressing, Malignant Solid Tumors	I	NCT01358903	Complete		CD44	No combination
		Acute Myelogenous Leukemia	I	NCT01641250	Complete			Cytarabine
Targeting Key Signals in ECM Remodeling	Dasatinib	Squamous Cell Lung Cancer	II	NCT01491633	Terminated	Dasatinib at a dose of 140 mg daily is associated with common toxicity and poor tolerability	DDR2	No combination
		Carcinoma, Non-Small-Cell Lung	II	NCT01514864	Terminated			No combination
	Merestinib	Breast Cancer	I	NCT03292536	Terminated		FAK	bisphosphonates or denosumab
	VS-6063	Ovarian Cancer	I/II	NCT03287271	Recruiting			carboplatin + paclitaxel
	BI 853520	Various Types of Advanced or Metastatic Cancer	I	NCT01335269	Complete	BI 853520 exhibits manageable, acceptable safety, favorable PK, and preliminary antitumor activity		No combination
	Nilotinib	Solid Tumors	II	NCT02029001	Recruiting			No combination

*ECM* Extracellular matrix, *PDAC* Pancreatic ductal adenocarcinoma, *HRT* Hypofractionated Radiotherapy, *LOXL2* Lysyl oxidase-like proteins 2, *HA* Hyaluronan, *CD44* Cluster of Differentiation 44, *OS* Overall Survival, *PFS* Progression-Free Survival, *RR* Response Rate, *TGF-β* Transforming growth factor β, *DDR2* Discoid domain receptor 2, *FAK* Focal adhesion kinase, *PK* Pharmacokinetics

### Targeting ECM components

#### Targeting collagen

Collagen occupies a central position in the composition of the ECM and is an important structural protein in both the BM and IM. During ECM remodeling, collagen provides a "barrier" for tumor drug resistance and immune suppression through deposition and cross-linking, and also offers specific "tracks" for tumor invasion and metastasis [381].

Animal studies have shown that the angiotensin receptor II inhibitor, losartan, can inhibit the production of collagen I and HA in CAFs, improve tumor stroma, and assist in the delivery of anti-tumor drugs [382, 383]. Losartan is currently being evaluated for its safety and efficacy in multiple clinical trials for cancer treatment (NCT01821729; NCT04106856). A Phase 2 clinical trial (NCT01821729) in patients with PDAC showed that neoadjuvant treatment with FOLFIRINOX combined with losartan prior to chemoradiotherapy increased the rate of margin-negative (R0) resection to 61% in 49 patients with previously unresectable PDAC, and 88% of those who underwent resection achieved R0 resection. This supports an anti-fibrotic strategy in PDAC to counteract the desmoplastic reaction and improve chemotherapeutic drug penetration into the tumor [384]. Furthermore, preclinical experiments involving losartan suggest that TGF-β is a key downstream factor through which losartan reduces stromal matrix production [382]. Furthermore, TGF-β reduces the density of the IM by decreasing Collagen I content, thereby enhancing the tissue penetration of doxorubicin into tumors [385]. Consequently, TGF-β may be a promising target for improving the ECM and assisting anti-tumor therapy. In a Phase I clinical trial, GC1008, a human anti-TGF-β monoclonal antibody, demonstrated acceptable safety in patients with malignant melanoma and renal cell carcinoma, and clinical benefit was observed in some patients, providing evidence supporting subsequent clinical trials (NCT00356460) [386]. Interim results from a Phase II clinical trial (NCT02688712) indicated that combining the TGF-β Type I receptor kinase inhibitor galunisertib with neoadjuvant chemoradiotherapy increased the complete response rate to 32% in patients with locally advanced rectal cancer, showing significant clinical benefit [387]. However, given the complex role of TGF-β and the TGF-β receptor in tumors, therapeutic strategies targeting them must be very cautious.

#### Targeting inhibition of collagen cross-linking

LOX is a key enzyme involved in the maturation and functional maintenance of collagen fibers in the ECM. LOX increases ECM stiffness and mechanical stability by catalyzing the covalent cross-linking of collagen and elastin [119]. Simtuzumab is a specific monoclonal antibody targeting LOXL2 intended to treat colorectal cancer and liver fibrosis by directly blocking the LOXL2-mediated catalytic activity for collagen cross-linking [337, 338]. However, results from a Phase II clinical trial (NCT01472198) showed that compared to placebo, gemcitabine combined with simtuzumab exhibited similar safety in patients with metastatic pancreatic cancer; the addition of simtuzumab did not improve patient survival (including progression-free survival and overall survival) or increase the objective response rate [388]. Although simtuzumab does not show clear clinical benefits, other drugs targeting LOX are actively being tested in clinical trials. For example, PAT-1251, which targets LOXL2, has shown good tolerability in a Phase I trial (NCT02852551).

#### Targeting HA

HA is also an important target for ECM-targeted therapy. A Phase II clinical trial demonstrated that the combination of PEGPH20 with nab-PTX and gemcitabine significantly improved the survival of patients with advanced pancreatic cancer, with superior efficacy observed in patients whose tumors had high HA expression (NCT01839487) [339]. A Phase III clinical trial evaluated the efficacy and safety of PEGPH20 plus nab-PTX and gemcitabine in patients with HA-high-metastatic PDAC (NCT02715804). However, results from an IB/II phase clinical trial of mFOLFIRINOX combined with the PEGPH20 regimen showed significantly increased toxicity and significantly shortened survival after combination with PEGPH20, leading to the cessation of patient recruitment for this study (NCT01959139) [389]. Furthermore, the combination of PEGPH20 with the PD-L1 inhibitor atezolizumab showed no clinical activity as a second-line treatment in patients with advanced PDAC or gastric adenocarcinoma who progressed during or after chemotherapy (NCT03193190; NCT03281369) [340]. Additionally, considering the critical role of the interaction between CD44 and HA in regulating the immune response and tumor progression [26], CD44 could be a potential target for cancer therapy. Significant progress has been achieved in numerous clinical trials targeting CD44. Among them, the CD44v6 antibody bivatuzumab mertansine was discontinued from clinical development due to its toxic side effects [390, 391]. Subsequently, additional CD44 antibodies, such as RO5429083 (NCT01358903; NCT01641250), have entered clinical trials.

### Targeting key signals in ECM remodeling

ECM remodeling is regulated by a complex network involving multiple factors and intertwined pathways. Among them, DDR, a collagen-specific receptor, regulates tumor cell survival and EMT upon activation [392]. FAK, an adhesion signaling "hub", mediates the transmission of ECM-integrin signals into cells, enhancing tumor cell invasiveness [393]. Based on these mechanisms, drugs targeting DDR and FAK are considered key strategies to overcome the vicious cycle of ECM-tumor interactions.

DDR1/2 is a receptor tyrosine kinase that specifically binds to collagen molecules and acts as an ECM signal transducer. It binds to ECM collagen and initiates intracellular signaling, promoting tumor proliferation, metastasis, and invasion among other processes [394]. In many cancers, DDR overexpression is associated with low overall survival rates [395, 396]. Therefore, DDR expression may be a potential biomarker and therapeutic target for cancer treatment. For example, DDR2 inhibitors (such as dasatinib) combined with PD-1 blockers can reduce the tumor burden [288]. Nilotinib inhibits cancer cells through the DDR signaling pathway [397]. The MET inhibitor merestinib (LY2801653) demonstrated good anti-tumor activity in mouse xenograft models and possessed potent inhibitory activity against DDR1/2. Thus, merestinib has potential clinical value in tumors carrying DDR1/2 mutations [398]. These drugs, including dasatinib (NCT01491633; NCT01514864), nilotinib (NCT02029001), and merestinib (NCT03292536), have been incorporated into multiple clinical trials. A drawback of these inhibitors is that they target not only DDR but also other receptor tyrosine kinases.

FAK is a non-receptor tyrosine kinase that includes FAK1 and PYK2/FAK2. FAK signaling plays an important role in stromal remodeling in the TME [399]. LOX-rich sEVs derived from CAFs mediate collagen cross-linking, which is a key upstream signal for FAK activation. Phosphorylation and activation of FAK drive tumor EMT and enhance tumor invasive capacity [121]. Furthermore, endogenous FAK in tumor cells contributes to the formation of a physical barrier by driving the formation of a dense fibrotic stroma and recruiting immunosuppressive cells, creating an immunosuppressive microenvironment. However, inhibiting FAK can reduce fibrosis and the immunosuppressive TME, potentially rendering previously unresponsive PDAC tumors responsive to chemotherapy and immunotherapy [400]. FAK inhibition has been identified as a potential adjuvant strategy for immunotherapy and chemotherapy. For example, clinical trials have investigated the use of the FAK inhibitor, defactinib, combined with carboplatin or PTX for resensitizing carboplatin-resistant ovarian cancer (NCT03287271). Additionally, numerous clinical trials have been established, such as a Phase I clinical trial evaluating the FAK inhibitor BI-853520 (IN10018) (NCT01335269).

## Conclusions and perspectives

Solid tumors are characterized by complex ECM architecture and marked intratumoral heterogeneity. The remodeled ECM extends beyond its conventional role as a structural scaffold, functioning not only to promote cell migration, tissue growth, and organ function, but also as a physical barrier, anchorage point, and signaling sensor, thereby accelerating cancer progression. The interplay and overlap between the ECM and immune cells integrate stromal biology, cancer biology, and immunology, providing a foundation for the development of novel cancer therapies. This review summarizes the pivotal role of ECM components in modulating anticancer immune responses and, conversely, how immune cells within the TME remodel the tumor ECM. The review further highlights the potential of targeting the tumor-associated ECM to enhance cancer immunotherapy. Although ECM-targeting strategies show promise for alleviating immunosuppression, their clinical translation remains challenging. Future research should prioritize the following key areas:

First, the spatiotemporal dynamics and heterogeneity of the ECM remain poorly understood. ECM composition, architecture, and mechanical properties vary significantly across tumor types, stages, and intratumoral regions. How such heterogeneity dynamically influences immune cell recruitment and functional states requires systematic investigation. Integrated multi-omics approaches, such as spatial transcriptomics coupled with proteomics combined with high-resolution mechanical imaging techniques (e. g., FRET-based stiffness sensors paired with SHG), could help construct spatiotemporal maps of ECM-immune cell interactions, enabling precise targets for personalized therapy.

Secondly, refined strategies for precise ECM modulation are required. Current ECM degradation or inhibition methods often cause nonspecific tissue damage or the release of pro-tumor factors and show limited efficacy in late-stage fibrotic tumors. Future efforts should focus on developing “smart-responsive” nanodelivery systems for the localized and controlled degradation of key ECM components (e. g., collagen and HA). Additionally, exploring combination therapies, such as ECM modulators with immune checkpoint inhibitors or CAR-T regimens, may help overcome stromal barriers and reprogram the immune microenvironment.

Finally, mechanistic studies on ECM-immune cell crosstalk should be expanded across multiple dimensions. Signaling networks downstream of ECM receptors in immune cell subsets remain incompletely mapped. Gene-edited organoid models and humanized mouse platforms can help elucidate how key receptor-mediated pathways influence immune exhaustion and memory formation. Furthermore, the CRISPR-based knockout of inhibitory ECM receptors in immune cells may enhance CAR-T/NK cell infiltration and cytotoxicity in dense stromal regions.

By addressing these challenges, future ECM-targeted approaches may evolve from merely disrupting the stroma to effectively reprogramming immunity, thereby opening new avenues for immunotherapy of solid tumors.

## Data Availability

Not applicable.

## Abbreviations

AIM2: Absent in Melanoma 2
ADAMs: A disintegrin and metalloproteinases
ARG1: Arginase-1
BAPN: β-Aminopropionitrile
BC: Breast cancer
BM: Basement membrane
BMDM: Bone marrow-derived macrophage
CAF: Cancer-associated fibroblast
CaP: Calcium phosphate
CAR: Chimeric antigen receptor
CCA: Cholangiocarcinoma
CCL21: C-C motif chemokine ligand 21
CCR2: C-X-C motif chemokine receptor 2
CD44: Cluster of Differentiation 44
CHI3L1: Chitinase 3-like Protein 1
Clever -1: Common lymphatic endothelial and vascular endothelial receptor-1
CSLC: Cancer stem-like cell
CTC: Circulating tumor cell
CTL: Cytotoxic T lymphocyte
CTLA-4: Cytotoxic T-Lymphocyte-Associated Protein 4
DC: Dendritic cell
DDR: Discoid domain receptor
dECM: Decellularized extracellular matrix
EC: Endothelial cell
ECM: Extracellular matrix
EDB: Extra-domain B
EGF: Epidermal growth factor
EBP: Elastin binding protein
ERK1/2: Extracellular Signal-Regulated Kinase1/2
EV: Extracellular vesicle
FAK: Focal adhesion kinase
FAP: Fibroblast activation protein
FBG: Fibrinogen-like globe
FcR: Fcg receptor
FN: Fibronectin
FRET: Förster resonance energy transfer
GAG: Glycosaminoglycan
GC: Gastric cancer
GFAT1: Glutamine-fructose aminotransferase 1
HA: Hyaluronan
HAPLN1: Hyaluronan and proteoglycan link protein 1
HBV: Hepatitis B
HCC: Hepatocellular carcinoma
HCV: Hepatitis C
HER2: Human epidermal growth factor receptor 2
HIF-1: Hypoxia-inducible factor 1
HMW-HA: High molecular weight HA
HPSE: Heparanase
HS: Hidradenitis suppurativa
iCAF: Inflammatory cancer associated fibroblast
ICB: Immune checkpoint blockade
IL-1β: Interleukin-1β
IM: Interstitial matrix
iNOS: Inducible nitric oxide synthase
ITAM: Immunoreceptor tyrosine-based activation motif
ITIM: Immunoreceptor tyrosine-based inhibition motif
LAIR: Leukocyte-associated Ig-like receptor
LGG: Low-grade glioma
LH2: Lysyl hydroxylase 2
LILRB4: Leukocyte immunoglobulin-like receptor B4
LMW-HA: Lower molecular weight HA
LOX: Lysyl oxidase
LOXL: Lysyl oxidase-like proteins
LUAD: Lung adenocarcinoma
MAPK: Mitogen-activated protein kinase
MAF: Metastasis associated fibroblast
MCP-1: Monocyte Chemoattractant Protein-1
MHC: Major histocompatibility complex
MIP-1α: Macrophage Inflammatory Protein-1 alpha
MMP: Matrix metalloproteinase
MoAb: Monoclonal antibody
MPM: Malignant pleural mesothelioma
mTOR: Mechanistic target of rapamycin
MyD88: Myeloid Differentiation Primary Response 88
NE: Neutrophil elastase
NET: Neutrophil extracellular traps
NF-κB: Nuclear factor-kappaB
NK: Natural killer
NP: Nanoparticle
NV: Nanovesicle
NSCLC: Non-small cell lung cancer
NSIM: Nonlinear stress inference microscopy
OPN: Osteopontin
OSCAR: Osteoclast-associated receptor
OSCC: Oral squamous cell carcinoma
OSM: Oncostatin M
PD-1: Programmed death 1
PDAC: Pancreatic ductal adenocarcinoma
PDGFR: Platelet-Derived Growth Factor Receptor
PD-L1: Programmed death-ligand 1
PSGL-1: P-selectin glycoprotein ligand-1
PTX: Paclitaxel
RA: Rheumatoid arthritis
ROS: Reactive oxygen species
SAXS-TT: Small-angle X-ray Scattering Tensor Tomography
SHG: Second-harmonic generation
SHP-1: Src homology 2 domain-containing protein tyrosine phosphatase-1
STAT3: Signal transducer and activator of transcription 3
TAM: Tumor-associated macrophages
TAN: Tumor-associated neutrophil
TCR: T-cell receptor
TFM: Traction force microscopy
TGF-β: Transforming growth factor-β
THP-1: Tamm-Horsfall Protein-1
TIM-3: T-cell immunoglobulin and mucin domain-containing protein-3
TIMP: Tissue inhibitors of metalloproteinases
TLR: Toll-like receptor
TME: Tumor microenvironment
TNBC: Triple-negative breast cancer
TNC: Tenascin-C
TNF-α: Tumor Necrosis Factor-α
Treg: Regulatory T cell
TRM: Tissue-resident macrophage
TSP-1: Thrombospondin-1
VEGF: Vascular endothelial growth factor
VLA: Very late antigen
XRF-CT: X-ray Fluorescence Computed Tomography
